# Genetic and environmental contributions to co-occurring physical health conditions in autism spectrum condition and attention-deficit/hyperactivity disorder

**DOI:** 10.1186/s13229-023-00548-3

**Published:** 2023-04-21

**Authors:** Pei-Yin Pan, Mark J. Taylor, Henrik Larsson, Catarina Almqvist, Paul Lichtenstein, Sebastian Lundström, Sven Bölte

**Affiliations:** 1 Center of Neurodevelopmental Disorders (KIND), Centre for Psychiatry Research; Department of Women’s and Children’s Health, Karolinska Institutet & Stockholm Health Care Services, Region Stockholm, Gävlegatan 22, 11330 Stockholm, Sweden; 2 Department of Medical Epidemiology and Biostatistics, Karolinska Institutet, Berzelius Väg 8, Solna, 17165 Stockholm, Sweden; 3 Lung and Allergy Unit, Astrid Lindgren Children’s Hospital, Karolinska University Hospital, Eugeniavägen 23, Solna, 17164 Stockholm, Sweden; 4 Gillberg Neuropsychiatry Centre, University of Gothenburg, Kungsgatan 12, 41119 GothenburgGöteborg, Sweden; 5 Centre for Ethics, Law, and Mental Health, University of Gothenburg, Universitetsplatsen 1, 41124 Gothenburg, Sweden; 6 Child and Adolescent Psychiatry, Stockholm Health Care Services, Region Stockholm, Solnavägen 1E, 113 65 Stockholm, Sweden; 7 Curtin Autism Research Group, Curtin School of Allied Health, Curtin University, Kent Street, Bentley, Perth, WA 6102 Australia

**Keywords:** Autism, ADHD, Twin study, Genetics, Comorbidity, Etiology

## Abstract

**Background:**

Autism spectrum condition and attention-deficit/hyperactivity disorder (ADHD) are associated with a range of physical health conditions. The aim of this study was to examine the etiological components contributing to co-occurring physical health conditions in autism and ADHD.

**Methods:**

In this nationwide Child and Adolescent Twin Study in Sweden, we analyzed data from 10,347 twin pairs aged 9 and 12. Clinical diagnoses of autism, ADHD, and physical health conditions were identified through the Swedish National Patient Register. Subclinical phenotypes of autism and ADHD were defined by symptom thresholds on a standardized parent-interview, the Autism–Tics, ADHD, and Other Comorbidities inventory. Associations between physical health conditions and autism**/**ADHD phenotypes were examined using generalized estimating equations. Bivariate twin models were applied to estimate the extent to which genetic and environmental risk factors accounted for physical health comorbidities.

**Results:**

Similar patterns of association with physical health conditions were found in clinical and subclinical autism/ADHD, with odds ratios ranging from 1.31 for asthma in subclinical ADHD to 8.03 for epilepsy in clinical autism. The estimated genetic correlation (*r*_*a*_) with epilepsy was 0.50 for clinical autism and 0.35 for subclinical autism. In addition, a modest genetic correlation was estimated between clinical autism and constipation (*r*_*a*_ = 0.31), functional diarrhea (*r*_*a*_ = 0.27) as well as mixed gastrointestinal disorders (*r*_*a*_ = 0.30). Genetic effects contributed 0.86 for mixed gastrointestinal disorders in clinical ADHD (*r*_*a*_ = 0.21). Finally, subclinical ADHD shared genetic risk factors with epilepsy, constipation, and mixed gastrointestinal disorders (*r*_*a*_ = 0.30, 0.17, and 0.17, respectively).

**Limitations:**

Importantly, since medical records from primary care were not included in the registry data used, we probably identified only more severe rather than the full range of physical health conditions. Furthermore, it needs to be considered that the higher prevalence of physical health conditions among autistic children and children with ADHD could be associated with the increased number of medical visits.

**Conclusions:**

Shared genetic effects contribute significantly to autism and ADHD phenotypes with the co-occurring physical health conditions across different organ systems, including epilepsy and gastrointestinal disorders. The shared genetic liability with co-occurring physical health conditions was present across different levels of autism and ADHD symptom severity.

**Supplementary Information:**

The online version contains supplementary material available at 10.1186/s13229-023-00548-3.

## Background

Autism spectrum condition (ASC) and attention-deficit/hyperactivity disorder (ADHD) are conditions of altered neurodevelopment caused by an interplay of polygenic predisposition and compounding environmental factors [1]. Autistic people or individuals with ADHD are at increased risk for physical health conditions across different body systems [2–5]. Recurrently highlighted co-occurring physical health conditions include epilepsy, immune dysregulation, and gastrointestinal (GI) dysfunction. The nature of the association between autism and ADHD behavioral phenotypes and these physical health conditions, and the potential shared etiological pathways has received growing scientific interest. Pediatric epilepsy is a condition of multiple genetic, cerebral, and metabolic etiologies and associated cognitive difficulties may be somewhat similar to those found in autism and ADHD [6]. Emerging evidence also suggests adverse effects of pre- and postnatal neuroinflammation on brain development and behavioral outcomes mimicking autism and ADHD [7, 8]. Finally, altered gut-brain axis mechanisms and dietary treatments are areas of topical interest in interventions aimed at autism and ADHD [9, 10].

While shared genetic susceptibility is postulated to partly account for the overlap between physical health conditions and autism/ADHD based on the identification of somatic pleiotropic genes [11], the magnitude of genetic contributions to comorbidity remains understudied. Five previous studies had examined genetic correlations between autism or ADHD and physical health conditions or physical/physiological parameters (e.g., asthma, migraine, height, triglycerides, fasting glucose) [12–16]. Three of them focused on adult ADHD or autism [12–14], and the other two explored childhood asthma and ADHD [15, 16]. However, these studies did not cover common co-occurring physical health conditions of clinical interest, such as epilepsy and pediatric GI disorders in childhood autism and ADHD. In addition, genetic contributions to autism and ADHD are likely to be distributed continuously in the general population [17], indicating gradually convergent patterns of co-occurring physical health conditions from behavioral traits to clinical diagnosis for these conditions. Therefore, a dichotomous classification of autism/ADHD by syndromic definition in previous study designs might have been of limited sensitivity to examine shared genetic effects with physical health conditions.

To unravel the genetic and environmental contributions to phenotypic correlations between two conditions/disorders with complex etiologies (i.e., autism/ADHD and physical health conditions), twin studies are particularly valuable and have been applied on the association between neurodevelopmental and physical health conditions [16]. While genome-wide association studies (GWAS) have started to estimate genetic correlations between autism/ADHD and physical health conditions [13], they were only based on common variants and have relatively low power yielding imprecise estimates.

Therefore, the objective of this study was to provide a comprehensive picture of common co-occurring physical health conditions in autistic children and children with ADHD at clinical and subclinical levels. We used information on clinical diagnoses and symptom severity of autism/ADHD from a population-based twin cohort to estimate the association of autism/ADHD phenotypes with physical health conditions. Moreover, we applied quantitative genetic modeling to estimate the degree to which co-occurring physical health conditions in clinical and subclinical autism/ADHD are accounted for by genetic and environmental factors. In this article, we adopted “identity-first” language (i.e., autistic individuals) rather than “person-first” language (i.e., individuals with autism) to avoid ableist language which could reflect discrimination and marginalization [18].

## Methods

### Participants

We used data collected from participants in the population-based Child and Adolescent Twin Study in Sweden (CATSS) [19]. This ongoing longitudinal study, initiated in 2004, invites all parents of twins aged 9 in Sweden to participate. (The earlier cohorts of CATSS included also twins aged 12.) The response rate was 70%. A total of 10,347 twin pairs were included in the current study, consisting of 8,125 nine-year-old and 2222 twelve-year-old twin pairs (4767 monozygotic [MZ] and 5580 same sex dizygotic [DZ] twin pairs). Zygosity was determined by a panel of 48 single-nucleotide polymorphisms (SNPs) or a validated questionnaire composed of 5 items on twin similarity. Zygosity was only assigned using the latter method if there was at least a 95% probability of correct classification. The study was approved by the Regional Ethical Review Board in Stockholm, Sweden.

### Diagnoses of autism, ADHD, and physical health conditions

Co-occurring physical health conditions [2, 3] were selected based on previous reports of associations with autism or ADHD and evidence of a strong genetic predisposition. Included physical health conditions were epilepsy, migraine, other headaches, allergic rhinitis, asthma, atopic dermatitis, allergy to specific allergen, coeliac disease, constipation, functional diarrhea, and irritable bowel syndrome [20–29]. Mixed headache (migraine and non-migraine headache) and mixed functional GI disorders (mixed FGIDs, including constipation, functional diarrhea, and irritable bowel syndrome) were also analyzed considering the ambiguous classification and idiopathic nature of these diagnoses [30, 31]. The diagnoses of autism, ADHD, and physical health conditions were identified by linking CATSS with the Swedish National Patient Register, containing medical records from in- and outpatient care. (Inpatient coverage is nationwide from 1987, and outpatient coverage is from 2001.) All diagnoses were defined based on International Classification of Diseases, Ninth Revision (ICD-9) and International Statistical Classification of Diseases and Related Health Problems, Tenth Revision (ICD-10) codes. The full list of diagnostic codes is given in Additional file 1.

### Subclinical phenotypes of autism and ADHD

Autism and ADHD symptoms were assessed with the autism (17 items) and ADHD modules (19 items) of the Autism–Tics, ADHD, and Other Comorbidities inventory (A-TAC), an open access instrument with full-versions in Swedish and English available on https://www.gu.se/en/gnc/gncs-resources/screening-questionnaires/a-tac-screening-questionnaire (also see Additional file 2 for the English version). The A-TAC is a standardized telephone interview with parents/caregivers as informants that has demonstrated good validity for screening the full range of neurodevelopmental disorder symptoms [32]. Each item in A-TAC is scored as 0 for “No,” 0.5 for “Yes, to some extent,” and 1 for “Yes.” The full A-TAC consists of 96 items, and the interview takes on average 32 min to administer. Subclinical phenotypes of autism were defined by scores of 4.5 or more on the A-TAC autism module (the lower cutoff value for screening autism, with sensitivity 0.85 and specificity 0.97, resulting in a prevalence of 3.6% screened positive in the general population); subclinical ADHD was defined by scores of at least 6.0 on the A-TAC ADHD module (the lower cutoff; sensitivity 0.79, specificity 0.90, resulting in a prevalence of 10.5% screened positive in the general population) [32].

### Statistical analysis

MZ and same-sex DZ twins were included in the analyses for the potential differences in variance components across sex. We did not model the effects of sex in our analysis since no sex-specific genetic influences on behavioral traits of autism/ADHD have been reported in the literature [33, 34]. Conditional multivariate logistic regression analysis with twin pairs clustered was used to explore the associations between physical health conditions and autism, ADHD, as well as the overlap of autism and ADHD by calculating odds ratios, adjusting for sex and age at measurement. Physical health conditions significantly associated with autism or ADHD phenotypes entered the bivariate twin analysis. Statistical significance was set at *P* < 0.05. Bonferroni correction was applied for multiple comparisons.

Structural equation modeling was used to estimate the relative genetic and environmental contributions to variation in liability to the clinical (categorical) phenotypes of a disorder, with the assumption that a continuous distribution of liability underlies such disorders. Liability variance was divided into three latent components: additive genetic (A), non-additive genetic (D) or shared environments (C), and nonshared environment (E). Only C or D can be estimated in a model, as they confound one another in the classical twin design. These components are estimated based on comparing the correlations between MZ twins who share all their segregating DNA and DZ twins who share on average 50% of their segregating genes. Principles of twin design are provided in detail elsewhere [35].

Univariate analyses were used to provide variance component estimates for physical health conditions and to test assumptions of the twin design. We tested only ACE but not ADE models to increase power. Thus, the proportion of genetic effects contributing to the variance should be interpreted as broad-sense heritability. Further nested models (AE and E models) were tested and compared to ACE models using the likelihood-ratio test. The correlations of one twin’s autism or ADHD phenotypes with their co-twin’s physical health condition (cross-twin cross-trait correlations) in MZ and DZ pairs were calculated. To study whether genetic and environmental risk factors for autism and ADHD phenotypes are associated with the co-occurring physical health conditions, we fitted the bivariate Cholesky decomposition to the categorical phenotype variables, which are presented here as the mathematically equivalent correlated factors solution. The genetic correlation estimates are correlation coefficients indicating the degree to which the genetic influences on two phenotypes correlate with one another. Bivariate heritability estimates, the proportion of phenotypic correlations explained by shared genetics, were calculated. To rule out that genetic correlations between subclinical autism/ADHD and co-occurring physical health conditions are driven by clinical autism/ADHD, sensitivity analyses were conducted by excluding clinical cases from subclinical groups. All twin models were conducted in OpenMx package for R [36].

## Results

The prevalence of autism, ADHD, and physical health conditions in our sample across and by sex/zygosity is presented in Table 1 (see Additional file 1: Table S1 for sex distribution and prevalence in different sex of autism/ADHD in Additional file 1). The prevalence of subclinical autism and subclinical ADHD in the sample for twin model analysis was 4.0% and 11.3%, respectively (please note that the physical health conditions in this study did not include the data from primary care health services). Table 2 summarizes the odds ratios (OR) of having physical health conditions in autism and ADHD for clinical and subclinical phenotypes. After adjusting for age and sex, both clinical and subclinical autism were associated with epilepsy, asthma, constipation, functional diarrhea, and mixed FGIDs. As for ADHD, clinical and subclinical phenotypes both displayed significant associations with epilepsy, asthma, constipation, and mixed FGIDs, while clinical ADHD was also associated with migraine and mixed headache. The ORs varied across different physical health conditions, ranging from OR = 1.31 for asthma in subclinical ADHD to OR = 8.03 for epilepsy in clinical autism. The associations between physical health conditions and the overlap of autism and ADHD as well as the mutually exclusive groups are shown in Additional file 1: Table S2.

**Table 1:** Prevalence of autism/ADHD and physical health conditions in the total sample and subsamples by sex/zygosity

Full sample (N)	Total		MZF		DZF		MZM		DZM		DZOS		Unknown		Sample for twin model analysis^a^	
	32,250		4,934		5,184		4,600		5,976		11,010		546		20,694	
	N	%	N	%	N	%	N	%	N	%	N	%	N	%	%	95% CI
Clinical autism	534	1.7	33	0.7	60	1.2	85	1.8	151	2.5	189	1.7	16	2.9	1.6	1.4, 1.8
Subclinical autism	1,251	3.9	96	1.9	157	3.0	211	4.6	328	5.5	434	3.9	25	4.6	4.0	3.7, 4.3
Clinical ADHD	1,427	4.4	117	2.4	169	3.3	240	5.2	369	6.2	501	4.6	31	5.7	4.3	4.0, 4.7
Subclinical ADHD	3,607	11.2	340	6.9	490	9.5	602	13.1	886	14.8	1,214	11.0	75	13.7	11.3	10.9, 11.8
Neurological disorders																
Epilepsy	392	1.2	57	1.2	56	1.1	51	1.1	92	1.5	128	1.2	8	1.5	1.2	1.1, 1.4
Migraine	522	1.6	81	1.6	106	2.0	58	1.3	102	1.7	171	1.6	4	0.7	1.7	1.5, 1.9
Other headache	267	0.8	55	1.1	50	1.0	26	0.6	33	0.6	100	0.9	3	0.5	0.8	0.7, 0.9
Mixed headache	730	2.3	126	2.6	144	2.8	80	1.7	127	2.1	247	2.2	6	1.1	2.3	2.1, 2.5
Immunological disorders																
Allergic rhinitis	1,920	6.0	244	4.9	234	4.5	301	6.5	445	7.4	670	6.1	26	4.8	5.9	5.6, 6.3
Asthma	4,317	13.4	514	10.4	695	13.4	598	13.0	960	16.1	1,476	13.4	74	13.6	13.4	12.8, 13.9
Atopic dermatitis	1,647	5.1	242	4.9	261	5.0	205	4.5	341	5.7	565	5.1	33	6.0	5.1	4.7, 5.4
Specific allergy	643	2.0	90	1.8	83	1.6	107	2.3	122	2.0	234	2.1	7	1.3	1.9	1.7, 2.2
Coeliac disease	362	1.1	72	1.5	73	1.4	29	0.6	52	0.9	131	1.2	5	0.9	1.1	0.9, 1.3
Gastrointestinal disorders																
Constipation	2,035	6.3	271	5.5	345	6.7	258	5.6	400	6.7	728	6.7	33	6.0	6.2	5.8, 6.5
Functional diarrhea	187	0.6	31	0.6	35	0.7	24	0.5	39	0.7	54	0.5	4	0.7	0.6	0.5, 0.7
Irritable bowel syndrome	127	0.4	30	0.6	25	0.5	7	0.2	11	0.2	53	0.5	1	0.2	0.4	0.3, 0.4
Mixed FGIDs	2,283	7.1	320	6.5	390	7.5	283	6.2	431	7.2	821	7.5	38	7.0	6.9	6.5, 7.3

The physical health conditions in this study did not include the data from primary care health services

*ADHD*, attention-deficit/hyperactivity disorder; *DZF*, dizygotic female; *DZM*, dizygotic male; *DZOS*, dizygotic opposite sex; *FGIDs*, functional gastrointestinal disorders; *MZF*, monozygotic female; *MZM*, monozygotic male

^a^MZ and same-sex DZ twins

**Table 2:** Association between autism/ADHD and physical health conditions in our sample (*N* = 20,964)

Co-occurring physical health conditions	Autism						ADHD					
	Clinical diagnosis			Subclinical phenotype			Clinical diagnosis			Subclinical phenotype		
	Odds ratio^a^	95% CI	*p*	Odds ratio^a^	95% CI	*p*	Odds ratio^a^	95% CI	*p*	Odds ratio^a^	95% CI	*p*
Neurological disorders												
** Epilepsy**	**8.03**	**5.32, 12.11**	** < 0.001**	**6.75**	**4.82, 9.45**	** < 0.001**	**3.52**	**2.46, 5.04**	** < 0.001**	**3.83**	**2.91, 5.06**	** < 0.001**
** Migraine**	0.75	0.28, 2.03	0.513	1.00	0.57, 1.74	0.985	**2.22**	**1.52, 3.25**	** < 0.001**	1.28	0.93, 1.76	0.126
Other headache	1.79	0.66, 4.87	0.258	1.06	0.46, 2.43	0.885	1.80	0.96, 3.35	0.066	1.21	0.73, 1.99	0.456
** Mixed headache**	1.13	0.56, 2.30	0.733	1.03	0.64, 1.65	0.911	**2.08**	**1.46, 2.97**	** < 0.001**	1.22	0.92, 1.62	0.166
Immunological disorders												
Allergic rhinitis	1.10	0.71, 1.72	0.665	1.01	0.74, 1.37	0.959	1.32	1.02, 1.71	0.037	1.05	0.87, 1.27	0.597
** Asthma**	**1.55**	**1.16, 2.08**	**0.003**	**1.45**	**1.23, 1.82**	** < 0.001**	**1.63**	**1.36, 1.96**	** < 0.001**	**1.31**	**1.16, 1.49**	** < 0.001**
Atopic dermatitis	1.28	0.78, 2.08	0.329	1.20	0.87, 1.65	0.264	1.52	1.15, 2.12	0.004	1.11	0.91, 1.36	0.303
Specific allergy	1.21	0.55, 2.65	0.644	0.93	0.54, 1.63	0.808	1.30	0.80, 2.12	0.294	0.87	0.62, 1.24	0.451
Coeliac disease	2.67	1.21, 5.85	0.015	1.45	0.75, 2.80	0.267	0.91	0.45, 1.83	0.784	1.24	0.81, 1.91	0.328
Gastrointestinal disorders												
** Constipation**	**2.03**	**1.43, 2.88**	** < 0.001**	**2.17**	**1.73, 2.72**	** < 0.001**	**1.91**	**1.53, 2.39**	** < 0.001**	**1.65**	**1.41, 1.93**	** < 0.001**
** Functional diarrhea**	**5.51**	**2.89, 10.52**	** < 0.001**	**2.67**	**1.48, 4.83**	**0.001**	2.13	1.15, 3.94	0.017	1.36	0.83, 2.25	0.226
Irritable bowel syndrome	2.25	0.55, 9.18	0.257	1.29	0.41, 4.11	0.667	1.58	0.57, 4.38	0.379	1.86	1.01, 3.42	0.047
** Mixed FGIDs**	**2.34**	**1.69, 3.24**	** < 0.001**	**2.08**	**1.67, 2.59**	** < 0.001**	**1.92**	**1.55, 2.38**	** < 0.001**	**1.59**	**1.37, 1.84**	** < 0.001**

The physical health conditions in this study did not include the data from primary care health services

*ADHD*, attention-deficit/hyperactivity disorder; *FGIDs*, functional gastrointestinal disorders

Statistical significance for this table was set at *p* < 0.0038 (Bonferroni correction for multiple comparisons)

Bold value: statistically significant and being included in bivariate twin model analysis

^a^Adjusted for age and sex

Cross-twin correlations and etiological components for each physical health disorder are provided in Table 3. The MZ twin correlations exceeded the DZ correlations for all the physical health conditions, indicating that the variation in liability to each condition was associated with genetic factors. Neurological conditions displayed heritability (*h*^*2*^) ranging from 0.41 for migraine to 0.61 for epilepsy, with no indication of shared environmental effects (see Additional file 1: Table S3 for model fit statistics). Immunological conditions, except for coeliac disease, showed similar heritability between 0.68 and 0.73. The proportions of etiological components for coeliac disease were different from those of others, in which A and C contributed to the etiology equally (0.49 and 0.47, respectively). As for GI conditions, genetics accounted for the variation in liability predominantly in three of the four conditions (range from 0.51 to 0.77), while the heritability of irritable bowel syndrome was estimated as 0.26.

**Table 3:** Univariate models for physical health conditions

Physical health conditions	Cross-twin correlation			The proportions explained by each etiology component						Heritability reported by other studies
				Genetic effects		Shared environmental effects		Nonshared environmental effects		
		*r*	95% CI	A/V	95% CI	C/V	95% CI	E/V	95% CI	*h*^2^
Neurological disorders										
Epilepsy	MZ	0.62	0.47, 0.75	0.61	0.24, 0.73	0		0.39	0.27, 0.54	range from 0.69 to 0.88 [20–22]
	DZ	0.25	0.04, 0.44							
Migraine	MZ	0.45	0.26, 0.60	0.41	0.07, 0.56	0		0.59	0.44, 0.75	0.45 (95% CI = 0.41, 0.49) [23] (meta-analysis)
	DZ	0.13	–0.06, 0.31							
Other headache	MZ	0.54	0.35, 0.70	0.53	0, 0.69	0		0.47	0.31, 0.66	range from 0.40 to 0.45 [23] (tension-type headache)
	DZ	0.18	–0.19, 0.47							
Mixed headache	MZ	0.49	0.36, 0.61	0.45	0.23, 0.57	0		0.55	0.43, 0.67	
	DZ	0.12	–0.05, 0.28							
Immunological disorders										
Allergic rhinitis	MZ	0.76	0.71, 0.81	0.73	0.55, 0.81	0.04	0, 0.19	0.24	0.19, 0.29	range from 0.33 to 0.91 [24]
	DZ	0.40	0.32, 0.47							
Asthma	MZ	0.83	0.80, 0.85	0.68	0.57, 0.79	0.15	0.05, 0.25	0.17	0.15, 0.20	0.54 (95% CI = 0.44, 0.63) [25] (meta-analysis)
	DZ	0.49	0.44, 0.54							
Atopic dermatitis	MZ	0.79	0.74, 0.83	0.73	0.55, 0.83	0.06	0, 0.22	0.21	0.20^a^, 0.26	0.74 (95% CI = 0.64, 0.83) [25] (meta-analysis)
	DZ	0.43	0.35, 0.50							
Specific allergy	MZ	0.90	0.85, 0.93	0.71	0.48, 0.93	0.19	0, 0.40	0.10	0.07, 0.14	0.63 (95% CI = 0.55, 0.71) [25] (meta-analysis)
	DZ	0.54	0.42, 0.65							
Coeliac disease	MZ	0.96	0.92, 0.98	0.49	0.30, 0.72	0.47	0.24, 0.65	0.04	0.02, 0.08	range from 0.57 to 0.87 [27–29]
	DZ	0.71	0.60, 0.80							
Gastrointestinal disorders										
Constipation	MZ	0.51	0.43, 0.59	0.51	0.30, 0.58	0		0.49	0.43, 0.49^a^	
	DZ	0.25	0.17, 0.33							
Functional diarrhea	MZ	0.93	0.86, 0.97	0.77	0.42, 0.97	0.16	0, 0.49	0.07	0.03, 0.14	
	DZ	0.54	0.33, 0.71							
Irritable bowel syndrome	MZ	0.69	0.46, 0.85	0.26	0, 0.83	0.43	0, 0.77	0.31	0.15, 0.53	0.22 (95% CI = 0.13, 0.30) [25] (meta-analysis)
	DZ	0.57	0.25, 0.79							
Mixed FGIDs	MZ	0.55	0.48, 0.62	0.56	0.39, 0.62	0		0.44	0.38, 0.51	0.57 (95% CI = 0.41, 0.76) [29]
	DZ	0.27	0.19, 0.34							

The physical health conditions in this study did not include the data from primary care health services

*A*, additive genetic variance; *C*, shared environmental variance; *DZ*, dizygotic twins; *E*, nonshared environmental variance; *FGIDs*, functional gastrointestinal disorders; *MZ*, monozygotic twins; *V*, phenotypic variance

^a^alpha level not reached

The co-occurrence rates and proband-wise cross-concordances for each physical health disorder associated with autism and ADHD are given in Table 4. Proband-wise cross-concordances denote the probability of co-twins of autism or ADHD probands having the diagnosis of each physical disorder. The MZ proband-wise cross-concordances were higher than DZ estimates for all GI disorders in clinical autism and functional diarrhea in subclinical autism, as well as epilepsy in both clinical and subclinical ADHD. Additional file 1: Table S4 in Additional file 1 presents the cross-twin cross-trait correlations between the associated physical health disorder and autism/ADHD. There were higher MZ than DZ cross-disorder correlations for all the physical health conditions in clinical autism, and for epilepsy, mixed headache, constipation, and mixed FGIDs in clinical ADHD, suggesting that genetic factors influence each condition and the covariance between them. However, regarding subclinical phenotypes of autism and ADHD, the MZ cross-disorder correlations were relatively equivalent to DZ estimates for all the physical health conditions.

**Table 4:** The co-occurrence rates and proband-wise cross-concordances for physical health conditions in autism and ADHD

Physical health conditions	Affected probands who also have physical health conditions				Proportion of co-twins with physical health conditions							
	Clinical diagnosis		Subclinical phenotype		Clinical diagnosis				Subclinical phenotype			
					MZ		DZ		MZ		DZ	
	%	95% CI	%	95% CI	%	95% CI	%	95% CI	%	95% CI	%	95% CI
*Autism*												
Neurological disorders												
Epilepsy	8.5	5.5, 11.5	4.8	3.1, 7.3	4.2	0.6, 7.9	4.3	1.5, 7.0	1.6	0.2, 3.0	2.3	1.0, 3.6
Immunological disorders												
Asthma	20.4	15.6, 25.1	21.6	17.8, 25.6	19.5	11.1, 27.8	22.3	16.4, 28.1	16.6	11.7, 21.5	16.5	13.0, 20.0
Coeliac disease	2.4	0.6, 4.3	1.5	0.7, 3.2	0.8	0, 2.5	2.4	0, 4.8	0.7	0, 1.6	1.2	0.1, 2.4
Gastrointestinal disorders												
Constipation	11.6	8.0, 15.1	13.8	10.8, 17.5	**11.0**	**4.8**, **17.2**	**8.1**	**4.4**, **11.7**	10.1	6.5, 13.7	10.9	8.2, 13.7
Functional diarrhea	3.0	1.2, 4.9	1.3	0.5, 2.9	**2.5**	**0**, **5.4**	**1.9**	**0.1**, **3.7**	**2.0**	**0.4, 3.5**	**1.0**	**0.1, 1.9**
Mixed FGIDs	14.3	10.4, 18.2	14.3	11.2, 18.1	**13.6**	**7.0**, **21.2**	**9.5**	**5.4**, **13.5**	11.1	7.3, 14.8	11.5	8.7, 14.3
*ADHD*												
Neurological disorders												
Epilepsy	3.9	2.6, 5.2	3.1	2.3, 4.3	**3.1**	**1.3**, **4.9**	**2.8**	**1.4**, **4.2**	**2.2**	**1.2, 3.2**	**1.9**	**1.1, 2.6**
Migraine	3.4	2.2, 4.5	1.9	1.3, 2.9	2.0	0.5, 3.4	3.7	2.1, 5.3	1.6	0.7, 2.4	2.0	1.2, 2.7
Mixed headache	4.2	2.9, 5.6	2.5	1.8, 3.6	3.4	1.2, 5.5	4.1	2.4, 5.7	2.1	1.1, 3.1	2.7	1.8, 3.5
Immunological disorders												
Asthma	20.7	17.8, 23.6	19.1	17.0, 21.5	20.7	15.9, 25.6	20.6	17.4, 24.2	16.7	13.8, 19.5	16.9	14.9, 19.0
Gastrointestinal disorders												
Constipation	10.7	8.7, 12.8	10.2	8.6, 12.1	8.7	5.5, 11.9	10.2	7.7, 12.7	7.1	5.3, 8.9	8.7	7.3, 10.2
Mixed FGIDs	11.8	9.7, 14.0	10.9	9.2, 12.8	10.6	7.2, 14.1	11.2	8.5, 13.8	8.3	6.4, 10.2	9.5	8.0, 11.0

The physical health conditions in this study did not include the data from primary care health services

*ADHD*, attention-deficit/hyperactivity disorder; *DZ*, dizygotic twins; *FGIDs*, functional gastrointestinal disorders; *MZ*, monozygotic twins

Bold values: Higher MZ than DZ estimates

**Fig. 1:**
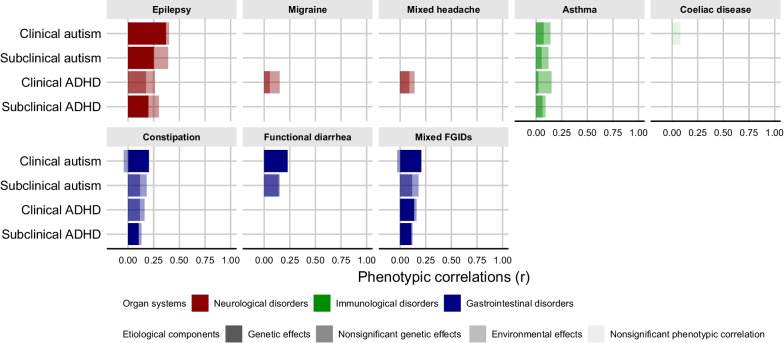
Etiological components contributing to phenotypic correlations between autism/ADHD and physical health conditions. *ADHD*, attention-deficit/hyperactivity disorder; *FGIDs*, functional gastrointestinal disorders

The phenotypic correlations, etiological correlations, and bivariate heritability between associated physical health conditions and autism/ADHD from each model are summarized in Fig. 1 and in Additional file 1: Table S5. Except for coeliac disease, there were significant phenotypic correlations (*rPH*) between all the physical health conditions and autism/ADHD for both clinical and subclinical phenotypes, ranging from 0.09 for asthma and subclinical ADHD to 0.40 for epilepsy and clinical autism. For autism, a common genetic liability was of major importance for co-occurring epilepsy in both clinical and subclinical phenotypes (proportions = 0.93 and 0.64, respectively). The estimated genetic correlation (*r*_*a*_) with epilepsy was 0.50 (95% CI = 0.27 − 0.76) for clinical autism and 0.35 (95% CI = 0.19 − 0.54) for subclinical autism. In addition, a modest genetic correlation was estimated between clinical autism and other physical health conditions, including constipation, functional diarrhea, and mixed FGIDs ([*r*_*a*_] as 0.27 [95% CI = 0.11 − 0.50], 0.31 [95% CI = 0.22 − 0.43], and 0.30 [95% CI = 0.12 − 0.63], respectively). For ADHD, subclinical phenotype displayed genetic correlations (*r*_*a*_) with epilepsy (0.30 [95% CI = 0.17 − 0.53]), constipation (0.17 [95% CI = 0.04 − 0.29]), and mixed FGIDs (0.17 [95% CI = 0.07 − 0.28]), while clinical ADHD showed significant genetic correlations (*r*_*a*_) only with mixed FGIDs (0.21 [95% CI = 0.02 − 0.44]). Model fit statistics are given in Additional file 1(Table S6-S9). Estimates derived from sensitivity analyses for subclinical autism/ADHD are presented in Additional file 1: Table S10.

## Discussion

To our knowledge, this is the first twin study to systematically examine the shared etiologies of common physical health conditions in children with clinical and subclinical autism and ADHD phenotypes. We found that both clinical and subclinical autism and ADHD displayed phenotypic correlations with neurological, immunological, and GI conditions, which were found also moderately to highly heritable. Our findings indicate that children with higher liability for clinical autism and ADHD have an increased risk for even various co-occurring physical health conditions, and that the risk extends to subclinical variants of autism and ADHD. In addition, the majority of co-occurring physical health conditions in autism and ADHD were explained by shared genetic factors. Taken together, the results of this study support the notion that autism and ADHD are quantitative extremes of continuously altered neurodevelopment, where cumulative genetic effects also contribute to an increased liability for co-occurring physical health conditions.

In addition to clinically diagnosed autism and ADHD, our findings suggest that subclinical phenotypes are also associated with a range of physical complications, with comorbidity profiles similar to those with clinical variants. Despite the under-threshold symptom severity, the potential clinical relevance of subclinical phenotypes of autism and ADHD has been discussed in the context of risk of psychiatric disorders and negative long-term outcomes [37–39]. Presently, little is known about co-occurring physical health conditions in subclinical manifestations of autism and ADHD. Physical health conditions have been linked to ADHD symptoms [40, 41], as well as to increased functional impairment and poorer clinical trajectories in clinical autism [42–45]. In addition, it has been reported that physical health conditions might play a role as the nonshared environmental factor contributing to the emergence of autism and ADHD symptoms among individuals with genetic predisposition [46, 47]. Based on the multifactorial model of autism and ADHD, the liability for clinical diagnosis is contingent on the accumulation and interplay of genetic and environmental risk factors [48, 49]. Individuals with subclinical variants, who have high levels of risk, are likely to be susceptible to develop symptoms meeting clinical diagnosis once the accumulated effects exceed the threshold [50, 51]. Therefore, the identification of potential indicators of subsequent autism/ADHD among the at-risk population might be clinically meaningful in terms of intervention. Our results highlight the need of further investigation on the impact and clinical implication of physical health conditions in subclinical autism and ADHD.

The univariate twin modeling analysis of epilepsy, coeliac disease, and the selected GI conditions was conducted with a child twin sample for the first time. The heritability of pediatric epilepsy and irritable bowel syndrome was found similar to the results derived from other adult samples [20–22, 25], while coeliac disease in children showed relatively lower heritability but higher proportions of common environmental variance [26–28]. It has been noted that there is a true rise of coeliac disease in recent decades, independent of the genetic background of the surveyed population [52]. The increased incidence of coeliac disease was attributed to emerging environmental elements which affected the immune tolerance to gluten in genetically predisposed children [53]. Our result might reflect the stronger role of environmental triggers contributing to coeliac disease among the young generation in contrast to adult cohorts. As for other FGIDs, despite some evidence on the familial aggregation [54, 55], the heritability of specific diseases has not been explored previously. The moderate to high heritability in our study suggests that genetic effects explain at least half of the liability variance to pediatric constipation and functional diarrhea.

Our results do not support an association between coeliac disease and clinical autism and ADHD. Although the potential benefit of gluten-free diet on symptoms of autism and ADHD has recently received considerable scientific attention [56–58], the epidemiological links are unclear. Positive associations have only been reported in large population-based samples with all manifestations of traits, subclinical and clinical forms of autism/ADHD, but not in smaller samples [59–62]. These discrepancies may reflect both autism and ADHD heterogeneity, and relatively small effect sizes of the associations with physical health conditions that could only be detected by samples with higher statistical power. Compared to the high co-occurrence of neurodevelopmental conditions [63], the comorbidity rates of physical health conditions in autism and ADHD were relatively low. Also, the phenotypic correlations between physical health conditions and autism as well as ADHD were low to moderate. Thus, we were unable to observe differences in MZ and DZ cross-concordances among the associated physical health conditions despite significant genetic correlations. These results emphasize the multifaceted nature and diverse somatic phenotypes of autism and ADHD, which could also reflect the disparity in treatment responses [9, 64]. Hence, it is recommended for future research to focus on defining the potential biological subtypes, the associated biomarkers, and the specific targeted interventions [65, 66].

The present study provides evidence that the risk for co-occurring physical health conditions increased with the higher liability to autism and ADHD diagnosis. These findings are consistent with a biomedical approach to autism and ADHD [2, 3], in light of somatic pleiotropy of genes associated with autism as well as ADHD [11]. The common etiology of neurodevelopmental conditions and physical health conditions might imply a broad phenotyping strategy in future genetic studies, in order to enhance gene discovery and the understanding of genetic architectures by phenotype-to-genotype approaches [67–70]. In addition, the continuous genetic effects on liability to co-occurring physical health conditions in autism and ADHD are in line with the concept of endophenotypes [71]. Therefore, genetically informative designs such as polygenic risk scores and linkage disequilibrium score regression might be applied to refined somatic phenotypes to aid in more straightforward and powerful genetic architecture analysis. Furthermore, an insight into the associated genetic variants for somatic phenotypes in autism/ADHD may facilitate the mapping of biological mechanisms onto behaviors, which is crucial for a molecular taxonomy [72], as well as the tailored pharmacological treatments for the specific subgroups [73].

On the other hand, the co-occurring physical health conditions in autism/ADHD could also be related to other factors than exclusively genetic liability. For instance, internalizing symptoms [74, 75], medications for behavior problems, including stimulants and antipsychotics, as well as behaviors such as food selectivity and toileting problems [76], have been shown to be associated with physical health. The same is true for healthcare access/quality, socioeconomic status, and lifestyle behaviors [77]. Therefore, a comprehensive assessment and awareness of the possibly environmentally related contributors to physical health are imperative for the adequate individualized support and intervention.

Our study had several strengths. First, we included both clinical diagnosis and subclinical phenotypes of autism and ADHD. The study design allowed us to examine the continuum model of genetics influence on co-occurring physical health conditions in autism and ADHD. Second, both autism and ADHD were analyzed in one cohort, resulting in a wealth of information on clinical and etiological profiles of somatic phenotypes in neurodevelopmental disorders.

## Limitations

However, our results should be interpreted taking into account some of the study’s limitations. The prevalence of headaches and GI conditions in our study could be underestimated compared to previous epidemiological studies which utilized systematic screening for data collection [31, 78]. Since the Swedish National Patient Register does not include the medical records in primary care, we probably only identified children with more severe physical diseases and thus the overall coverage of physical health conditions across the full range of severity might be incomplete. There is also a possibility that the increased number of physical health conditions detected in autistic children or children with ADHD is associated with their increased number of medical visits [79–82]. In addition, our results may also not generalize beyond children in light of the differences in nature and profiles of physical health conditions between children and adults. For example, congenital structural alterations are usually present in epilepsy which develops in childhood, while head trauma, infection, and brain tumors might cause epilepsy at any age [83]. Also, pediatric headache phenotypes are difficult to classify and could continuously evolve into adulthood [84]. Future work should seek to assess whether the etiology of co-occurring physical health conditions in autism and ADHD changes across the life span. Moreover, we did not include other demographic characteristics in the analysis of the association between physical health conditions and autism/ADHD, such as geographic distribution, socioeconomic status, and ethnicity, which might confound our results [2, 3]. The aforementioned demographic variables were not available in our dataset, and thus, we are unable to determine how representative this sample is. Furthermore, the prevalence of autism/ADHD and physical health conditions could influence the estimates of heritability [85, 86]. Therefore, the generalizability of our results to countries outside Northern Europe should be considered. Other factors which could impact the representativeness of our sample include non-response bias (response rate 70%), the sampling (to sample only twins within a population), and that twins have increased risk for low birth weight, which is associated with neurodevelopmental and physical health conditions [87–89]. Finally, children with co-occurring autism and ADHD tended to have similar risks for physical health conditions to those with autism or ADHD in our sample, for both clinical diagnosis and subclinical phenotypes. Nevertheless, we were unable to examine the complete profile of physical health conditions in this group as well as the mutually exclusive samples of autism and ADHD due to limited statistical power. In view of the shared genetic liability between autism and ADHD [63], it could be of value to explore in more detail and a larger sample the manifestation and etiology of physical health conditions in individuals with “exclusive autism,” “exclusive ADHD,” and “overlapping of autism and ADHD” in future research.

## Conclusions

This study endorses the quantitative nature of autism and ADHD with genetic effects forming a continuum of behavioral traits that are associated with physical health conditions in different body systems. These associations vary in strength, and further research into the genetic links, biological pathways, and clinical implications of somatic phenotypes are required for a more complete picture of physical health in autism and ADHD.

## Supplementary Information

**Additional file 1.** ICD code list. Table S1: Sex distribution of clinical and subclinical autism/ADHD in our sample. Table S2: Associations between physical health conditions and the overlap of autism and ADHD in our sample. Table S3: Univariate twin model fit statistics. Table S4: Bivariate models for autism/ADHD and physical health conditions. Table S5: Etiological component contributing to phenotypic correlation between autism/ADHD and physical health conditions. Table S6: Bivariate twin model of clinical autism and physical health conditions fit statistics. Table S7: Bivariate twin model of subclinical autism and physical health conditions fit statistics. Table S8: Bivariate twin model of clinical ADHD and physical health conditions fit statistics. Table S9: Bivariate twin model of subclinical ADHD and physical health conditions fit statistics. Table S10: Etiological component contributing to phenotypic correlation between subclinical autism/ADHDand physical health conditions.

**Additional file 2.** The English version of the Autism–Tics, ADHD, and Other Comorbidities inventory (A-TAC).

## Data Availability

The Public Access to Information and Secrecy Act in Sweden prohibits us from making individual-level data publicly available. Researchers who are interested in replicating our work can apply for individual-level data through The Child and Adolescent Twin Study in Sweden (CATSS) at: https://ki.se/en/meb/the-child-and-adolescent-twin-study-in-sweden-catss.

## Abbreviations

ASC: Autism spectrum condition
ADHD: Attention-deficit/hyperactivity disorder
GI: Gastrointestinal
GWAS: Genome-wide association studies
CATSS: Child and Adolescent Twin Study in Sweden
MZ: Monozygotic
DZ: Dizygotic
SNP: Single-nucleotide polymorphisms
FGIDs: Functional gastrointestinal disorders
ICD-9: International Classification of Diseases, Ninth Revision
ICD-10: International Statistical Classification of Diseases and Related Health Problems, Tenth Revision
A-TAC: The Autism-Tics, ADHD, and Other Comorbidities inventory
OR: Odds ratios
