# Supplementary material for: Genetic and environmental contributions to co-occurring physical health conditions in autism spectrum condition and attention-deficit/hyperactivity disorder
Source: Mol Autism. 2023 Apr 21;14:17. doi: 10.1186/s13229-023-00548-3 (PMC10122407; doi:10.1186/s13229-023-00548-3)
Supplement: Supplementary file 1 — Additional file 1. ICD code list. Table S1: Sex distribution of clinical and subclinical autism/ADHD in our sample. Table S2: Associations between physical health conditions and the overlap of autism and ADHD in our sample. Table S3: Univariate twin model fit statistics. Table S4: Bivariate models for autism/ADHD and physical health conditions. Table S5: Etiological component contributing to phenotypic correlation between autism/ADHD and physical health conditions. Table S6: Bivariate twin model of clinical autism and physical health conditions fit statistics. Table S7: Bivariate twin model of subclinical autism and physical health conditions fit statistics. Table S8: Bivariate twin model of clinical ADHD and physical health conditions fit statistics. Table S9: Bivariate twin model of subclinical ADHD and physical health conditions fit statistics. Table S10: Etiological component contributing to phenotypic correlation between subclinical autism/ADHDand physical health conditions. [file 13229_2023_548_MOESM1_ESM.pdf]

Supplementary Information

ICD code list.

Categories	Disorders	ICD-10	ICD-9
Neurodevelopmental disorders	Autism	F84.0, F84.5, F84.9	299.0, 299.8, 299.9
	ADHD	F90	314.0
Neurological disorders	Epilepsy	G40, G41	345
	Migraine	G43	346
	Headache	G44	784.0
Immunological disorders	Asthma	J45	493
	Allergic rhinitis	J30	477
	Atopic dermatitis	L20	691.8
	Allergy to any allergens	Z91.0	995.3
	Coeliac disease	K90.0	579.0
Gastrointestinal disorders	Irritable bowel syndrome	K58	564.1
	Constipation	K59.0	564.0
	Functional diarrhea	K59.1	564.5

Abbreviations: ADHD, attention-deficit/hyperactivity disorder; ICD-9, International Classification of Diseases, Ninth Revision; ICD-10, International Statistical Classification of Diseases and Related Health Problems, Tenth Revision

Table S1. Sex distribution of clinical and subclinical autism/ADHD in our sample (N=20,964)

	Autism				ADHD				Autism and ADHD			
	Clinical diagnosis N=329		Subclinical phenotype N=792		Clinical diagnosis N=895		Subclinical phenotype N=2,318		Clinical diagnosis N=155		Subclinical phenotype N=605	
	Male N=236	Female N=93	Male N=539	Female N=253	Male N=609	Female N=286	Male N=1,488	Female N=830	Male N=117	Female N=38	Male N=416	Female N=189
Sex distribution	71.7%	28.3%	68.1%	31.9%	68.0%	32.0%	64.2%	35.8%	75.5%	24.5%	68.9%	31.2%
Prevelance in different sex	2.2%	0.9%	5.1%	2.5%	5.8%	2.8%	14.1%	8.2%	1.1%	0.4%	3.9%	1.9%

	Autism only (ADHD excluded)				ADHD only (autism excluded)			
	Clinical diagnosis N=174		Subclinical phenotype N=187		Clinical diagnosis N=740		Subclinical phenotype N=1,713	
	Male N=119	Female N=55	Male N=123	Female N=64	Male N=492	Female N=248	Male N=1,072	Female N=641
Sex distribution	68.4%	31.6%	65.8%	34.2%	66.5%	33.5%	62.6%	37.4%
Prevelance in different sex	1.1%	0.5%	1.2%	0.6%	4.7%	2.5%	10.1%	6.3%

Table S2. Associations between physical health conditions and the overlap of autism and ADHD in our sample (N=20,964)

	Autism only (ADHD excluded)				ADHD only (autism excluded)				Autism and ADHD			
	Clinical diagnosis N=174		Subclinical phenotype N=187		Clinical diagnosis N=740		Subclinical phenotype N=1,713		Clinical diagnosis N=155		Subclinical phenotype N=605	
Co-occurring physical health conditions	Odds ratio[a]	*p*	Odds ratio[a]	*p*	Odds ratio[a]	*p*	Odds ratio[a]	*p*	Odds ratio[a]	*p*	Odds ratio[a]	*p*
Neurological disorders												
**Epilepsy**	**10.06**	**<0.001**	**6.55**	**<0.001**	**3.03**	**<0.001**	**2.31**	**<0.001**	**4.96**	**<0.001**	**5.24**	**<0.001**
**Migraine**	0.69	0.608	0.32	0.257	**2.50**	**<0.001**	1.29	0.169	0.82	0.776	1.24	0.461
Other headache	2.40	0.139	0.72	0.747	1.94	0.047	1.23	0.485	0.99	0.994	1.18	0.717
**Mixed headache**	1.30	0.566	0.47	0.292	**2.31**	**<0.001**	1.22	0.236	0.93	0.895	1.23	0.416
Immunological disorders												
Allergic rhinitis	0.81	0.534	0.41	0.049	1.29	0.088	1.00	0.997	1.44	0.224	1.23	0.213
**Asthma**	1.35	0.144	1.24	0.293	**1.59**	**<0.001**	1.20	0.014	1.76	0.008	**1.59**	**<0.001**
Atopic dermatitis	1.05	0.895	0.73	0.422	1.51	0.010	1.03	0.832	1.52	0.200	1.36	0.082
Specific allergy	0.87	0.810	0.80	0.710	1.23	0.453	0.86	0.459	1.56	0.408	0.98	0.939
Coeliac disease	2.96	0.019	0.52	0.519	0.67	0.373	1.05	0.855	2.22	0.170	1.76	0.112
Gastrointestinal disorders												
**Constipation**	**2.33**	**<0.001**	1.57	0.078	**1.93**	**<0.001**	**1.33**	**0.003**	1.69	0.052	**2.34**	**<0.001**
**Functional diarrhea**	**5.14**	**<0.001**	1.91	0.417	1.35	0.468	0.829	0.580	**5.48**	**<0.001**	**2.87**	**0.001**
Irritable bowel syndrome	3.98	0.056	NA	NA	1.87	0.229	1.83	0.087	NA	NA	1.78	0.328
**Mixed FGIDs**	**2.66**	**<0.001**	1.49	0.107	**1.87**	**<0.001**	1.29	0.005	1.97	0.005	**2.25**	**<0.001**

Physical health conditions in this study did not include the data from primary care health services.
Abbreviations: ADHD, attention-deficit/hyperactivity disorder; FGIDs, functional gastrointestinal disorders
Statistical significance for this table was set at $p<0.0038$ (Bonferroni correction for multiple comparisons)
Bold value: statistically significant and being included in bivariate twin model analysis
[a]adjusted for age and sex

Table S3. Univariate twin model fit statistics

Model		−2LL	Parameters	*df*	AIC	Comparison model	Δ$\chi 2$	Δ*df*	*p*
Neurological disorders									
	Epilepsy								
	Fully saturated	2703.30	6	20688	-38672.71	NA	NA	NA	NA
	Eq thresh across twins	2703.55	4	20690	-38676.45	Fully saturated	0.26	2	0.879
	Eq thresh across zygosity	2704.95	3	20691	-38677.05	Fully saturated	1.66	3	0.647
	ACE	2705.25	4	20692	-38678.75	Fully saturated	1.96	4	0.743
	AE	2705.25	3	20693	-38680.75	ACE	0.00	1	1.000
	CE	2714.15	3	20693	-38671.85	ACE	8.89	1	0.003
	E	2757.74	2	20694	-38630.26	ACE	52.49	2	<0.001
	Migraine								
	Fully saturated	3497.39	6	20688	-37878.61	NA	NA	NA	NA
	Eq thresh across twins	3497.90	4	20690	-37882.10	Fully saturated	0.51	2	0.776
	Eq thresh across zygosity	3502.74	3	20691	-37879.26	Fully saturated	5.35	3	0.148
	ACE	3503.51	4	20692	-37880.50	Fully saturated	6.12	4	0.191
	AE	3503.50	3	20693	-37882.50	ACE	0.00	1	1.000
	CE	3508.59	3	20693	-37877.41	ACE	5.08	1	0.024
	E	3525.41	2	20694	-37862.59	ACE	21.91	2	<0.001
	Headache								
	Fully saturated	1884.60	6	20688	-39491.40	NA	NA	NA	NA
	Eq thresh across twins	1887.08	4	20690	-39492.92	Fully saturated	2.48	2	0.290
	Eq thresh across zygosity	1887.75	3	20691	-39494.25	Fully saturated	3.15	3	0.369
	ACE	1888.05	4	20692	-39495.95	Fully saturated	3.45	4	0.486
	AE	1888.05	3	20693	-39497.95	ACE	0.00	1	1.000
	CE	1891.87	3	20693	-39494.13	ACE	3.83	1	0.050
	E	1913.47	2	20694	-39474.53	ACE	25.43	2	<0.001
	Mixed headache								
	Fully saturated	4491.45	6	20688	-36884.55	NA	NA	NA	NA
	Eq thresh across twins	4491.84	4	20690	-36888.16	Fully saturated	0.40	2	0.819

	Eq thresh across zygosity	4493.35	3	20691	-36888.65	Fully saturated	1.90	3	0.593
	ACE	4495.45	4	20692	-36888.55	Fully saturated	4.01	4	0.405
	AE	4495.45	3	20693	-36890.55	ACE	0.00	1	1.000
	CE	4506.02	3	20693	-36879.98	ACE	10.56	1	0.001
	E	4539.58	2	20694	-36848.42	ACE	44.12	2	<0.001
Immunological disorders									
	Allergic rhinitis								
	Fully saturated	8780.31	6	20688	-32595.70	NA	NA	NA	NA
	Eq thresh across twins	8781.87	4	20690	-32598.13	Fully saturated	1.56	2	0.458
	Eq thresh across zygosity	8782.83	3	20691	-32599.18	Fully saturated	2.52	3	0.472
	ACE	8782.83	4	20692	-32601.17	Fully saturated	2.52	4	0.641
	AE	8783.05	3	20693	-32602.95	ACE	0.22	1	0.636
	CE	8848.09	3	20693	-32537.91	ACE	65.27	1	<0.001
	E	9296.39	2	20694	-32091.61	ACE	513.56	2	<0.001
	Asthma								
	Fully saturated	14930.06	6	20688	-26445.94	NA	NA	NA	NA
	Eq thresh across twins	14934.51	4	20690	-26445.49	Fully saturated	4.45	2	0.108
	Eq thresh across zygosity	14939.62	3	20691	-26442.39	Fully saturated	9.55	3	0.023
	ACE	14939.62	4	20692	-26444.39	Fully saturated	9.55	4	0.049
	AE	14947.87	3	20693	-26438.14	ACE	8.25	1	0.004
	CE	15089.09	3	20693	-26296.91	ACE	149.47	1	<0.001
	E	16281.19	2	20694	-25106.81	ACE	1341.57	2	<0.001
	Atopic dermatitis								
	Fully saturated	7800.21	6	20688	-33575.79	NA	NA	NA	NA
	Eq thresh across twins	7800.24	4	20690	-33579.76	Fully saturated	0.04	2	0.981
	Eq thresh across zygosity	7804.28	3	20691	-33577.72	Fully saturated	4.08	3	0.253
	ACE	7804.28	4	20692	-33579.72	Fully saturated	4.08	4	0.396
	AE	7804.89	3	20693	-33581.11	ACE	0.60	1	0.437
	CE	7867.12	3	20693	-33518.88	ACE	62.83	1	<0.001
	E	8300.15	2	20694	-33087.85	ACE	495.87	2	<0.001

	Specific allergy								
	Fully saturated	3540.12	6	20688	-37835.88	NA	NA	NA	NA
	Eq thresh across twins	3542.96	4	20690	-37837.04	Fully saturated	2.85	2	0.241
	Eq thresh across zygosity	3543.99	3	20691	-37838.01	Fully saturated	3.87	3	0.276
	ACE	3543.99	4	20692	-37840.01	Fully saturated	3.87	4	0.424
	AE	3546.18	3	20693	-37839.82	ACE	2.19	1	0.139
	CE	3588.77	3	20693	-37797.23	ACE	44.79	1	<0.001
	E	3964.82	2	20694	-37423.18	ACE	420.84	2	<0.001
	Coeliac disease								
	Fully saturated	2105.49	6	20688	-39270.51	NA	NA	NA	NA
	Eq thresh across twins	2108.70	4	20690	-39271.30	Fully saturated	3.20	2	0.202
	Eq thresh across zygosity	2108.81	3	20691	-39273.19	Fully saturated	3.32	3	0.345
	ACE	2108.81	4	20692	-39275.19	Fully saturated	3.32	4	0.506
	AE	2122.20	3	20693	-39263.80	ACE	13.39	1	<0.001
	CE	2138.18	3	20693	-39247.82	ACE	29.36	1	<0.001
	E	2491.24	2	20694	-38896.76	ACE	382.42	2	<0.001
Gastrointestinal disorders									
	Constipation								
	Fully saturated	9401.29	6	20688	-31974.71	NA	NA	NA	NA
	Eq thresh across twins	9401.84	4	20690	-31978.16	Fully saturated	0.56	2	0.757
	Eq thresh across zygosity	9411.64	3	20691	-31970.36	Fully saturated	10.36	3	0.016
	ACE	9411.65	4	20692	-31972.35	Fully saturated	10.37	4	0.035
	AE	9411.65	3	20693	-31974.35	ACE	0.00	1	1.000
	CE	9431.85	3	20693	-31954.15	ACE	20.20	1	<0.001
	E	9570.92	2	20694	-31817.08	ACE	159.27	2	<0.001
	Functional diarrhea								
	Fully saturated	1403.84	6	20688	-39972.17	NA	NA	NA	NA
	Eq thresh across twins	1405.18	4	20690	-39974.82	Fully saturated	1.35	2	0.510
	Eq thresh across zygosity	1405.63	3	20691	-39976.37	Fully saturated	1.79	3	0.616
	ACE	1405.62	4	20692	-39978.37	Fully saturated	1.79	4	0.774

	AE	1406.22	3	20693	-39979.78	ACE	0.59	1	0.442
	CE	1427.34	3	20693	-39958.66	ACE	21.71	1	<0.001
	E	1567.26	2	20694	-39820.74	ACE	161.63	2	<0.001
	Irritable bowel syndrome								
	Fully saturated	925.60	6	20688	-40450.40	NA	NA	NA	NA
	Eq thresh across twins	932.77	4	20690	-40447.23	Fully saturated	7.17	2	0.028
	Eq thresh across zygosity	933.31	3	20691	-40448.69	Fully saturated	7.71	3	0.052
	ACE	933.31	4	20692	-40450.69	Fully saturated	7.71	4	0.103
	AE	935.17	3	20693	-40450.83	ACE	1.86	1	0.173
	CE	933.86	3	20693	-40452.14	ACE	0.546	1	0.460
	E	970.22	2	20694	-40417.78	ACE	36.91	2	<0.001
	Mixed FGIDs								
	Fully saturated	10135.37	6	20688	-31240.63	NA	NA	NA	NA
	Eq thresh across twins	10136.54	4	20690	-31243.47	Fully saturated	1.17	2	0.558
	Eq thresh across zygosity	10143.76	3	20691	-31238.25	Fully saturated	8.39	3	0.039
	ACE	10143.91	4	20692	-31240.09	Fully saturated	8.54	4	0.074
	AE	10143.91	3	20693	-31242.09	ACE	0.00	1	1.000
	CE	10175.61	3	20693	-31210.40	ACE	31.69	1	<0.001
	E	10370.00	2	20694	-31018.00	ACE	226.08	2	<0.001

Physical health conditions in this study did not include the data from primary care health services.

Abbreviations: −2LL, −2*log-likelihood; A, additive genetic component; AIC, Akaike Information Criteria; C, shared environmental component; E, nonshared environmental component; Eq, equate; Mixed FGIDs, mixed functional gastrointestinal disorders; NA, not applicable

Table S4. Bivariate models for autism/ADHD and physical health conditions

Co-occurring physical health conditions			Cross-trait cross-twin correlations							
			Autism				ADHD			
			Clinical autism		Subclinical autism		Clinical ADHD		Subclinical autism	
			r	95% CI	*r*	95% CI	*r*	95% CI	*r*	95% CI
Neurological disorders										
	Epilepsy	MZ	**0.39**	**0.28, 0.45**	**0.10**	**0.10, 0.41**	**0.17**	**0.08, 0.25**	-0.03	-0.09, 0.04
		DZ	**0.23**	**0.11, 0.35**	**0.09**	**0.07, 0.10**	0.09	-0.01, 0.19	**-0.09**	**-0.09, -0.03**
	Migraine	MZ					0.08	-0.03, 0.19		
		DZ					0.09	-0.00, 0.19		
	Headache	MZ								
		DZ								
	Mixed headache	MZ					**0.10**	**0.00, 0.20**		
		DZ					0.08	-0.01, 0.17		
Immunological disorders										
	Allergic rhinitis	MZ								
		DZ								
	Asthma	MZ	**0.16**	**0.15, 0.16**	-0.03	-0.09, 0.03	0.05	-0.00, 0.10	**0.08**	**0.03, 0.13**
		DZ	**0.04**	**0.04, 0.05**	-0.05	-0.10, 0.01	0.05	-0.00, 0.10	**0.07**	**0.03, 0.12**
	Atopic dermatitis	MZ								
		DZ								
	Specific allergy	MZ								
		DZ								
	Coeliac disease	MZ	**0.10**	**0.10, 0.24**						
		DZ	0.07	-0.10, 0.23						
Gastrointestinal disorders										
	Constipation	MZ	**0.07**	**0.06, 0.13**	**0.12**	**0.04, 0.19**	**0.15**	**0.08, 0.22**	0.06	-0.01, 0.12
		DZ	**-0.04**	**-0.06, -0.01**	**0.12**	**0.05, 0.19**	**0.10**	**0.04, 0.17**	0.05	-0.01, 0.10
	Functional diarrhea	MZ	**0.24**	**0.10, 0.38**	0.03	-0.04, 0.13				
		DZ	**0.11**	**0.08, 0.28**	0.00	-0.07, 0.02				

	Irritable bowel syndrome	MZ								
		DZ								
	Mixed FGIDs	MZ	**0.08**	**0.06, 0.08**	**0.10**	**0.02, 0.11**	**0.16**	**0.09, 0.22**	**0.08**	**0.02, 0.14**
		DZ	-0.04	-0.12, 0.01	**0.11**	**0.04, 0.18**	**0.10**	**0.03, 0.17**	**0.05**	**0.00, 0.11**

Physical health conditions in this study did not include the data from primary care health services.
Bold values: statistically significant
Shading: bivariate twin model analysis not performed

Table S5. Etiological component contributing to phenotypic correlation between autism/ADHD and physical health conditions

Co-occurring physical health conditions	Clinical diagnosis: Phenotypic Correlation *r*	Clinical diagnosis: Phenotypic Correlation 95% CI	Clinical diagnosis: Etiological components	Clinical diagnosis: Correlations *r*	Clinical diagnosis: Correlations 95% CI	Clinical diagnosis: Propor-tions	Subclinical phenotype: Phenotypic Correlation *r*	Subclinical phenotype: Phenotypic Correlation 95% CI	Subclinical phenotype: Etiological components	Subclinical phenotype: Correlations *r*	Subclinical phenotype: Correlations 95% CI	Subclinical phenotype: Propor-tions
Autism												
Neurological disorders												
Epilepsy	**0.40**	**0.33, 0.49**	**A**	**0.50**	**0.27, 0.76**	**0.93**	**0.39**	**0.31, 0.46**	**A**	**0.35**	**0.19, 0.54**	**0.64**
			C	1.00	-1.00, 1.00	0.00			C	0.96	-1.00, 0.99	0.00
			E	0.16	-0.26, 0.51	0.07			E	0.55	0.44, 0.95	0.36
Immunological disorders												
Asthma	**0.14**	**0.07, 0.20**	A	0.09	-0.08, 0.28	0.53	**0.12**	**0.07, 0.17**	A	0.07	-0.05, 0.15	0.47
			C	1.00	-1.00, 1.00	0.62			C	1.00	-0.28, 1.00	0.15
			E	-0.16	-0.47, 0.21	-0.15			E	0.25	0.03, 0.53	0.38
Coeliac disease	0.08	-0.02, 0.27	A	0.03	-0.12, 0.46	0.32						
			C	1.00	-1.00, 1.00	0.54						
			E	0.23	-0.47, 1.00	0.15						
Gastrointestinal disorders												
Constipation	**0.16**	**0.15, 0.23**	**A**	**0.31**	**0.22, 0.43**	**1.26**	**0.18**	**0.13, 0.23**	A	0.20	-0.04, 0.39	0.66
			C	-1.00	-1.00, 1.00	-0.12			C	1.00	-1.00, 1.00	0.18
			E	-0.11	-0.47, 0.20	-0.14			E	0.09	-0.12, 0.31	0.15
Functional diarrhea	**0.23**	**0.10, 0.34**	**A**	**0.27**	**0.11, 0.50**	**1.00**	**0.15**	**0.04, 0.26**	A	0.17	-0.10, 0.47	0.95
			C	0.62	-1.00, 0.65	0.00			C	0.97	-0.90, 1.00	0.04
			E	0.00	-0.99, 1.00	0.00			E	0.03	-0.34, 0.73	0.01
Mixed FGIDs	**0.18**	**0.11, 0.25**	**A**	**0.30**	**0.12, 0.63**	**1.15**	**0.17**	**0.12, 0.23**	A	0.18	-0.03, 0.34	0.69
			C	-1.00	-1.00, -1.00	-0.05			C	1.00	-1.00, 1.00	0.14
			E	-0.09	-0.46, 0.35	-0.10			E	0.11	-0.11, 0.32	0.17
ADHD												
Neurological disorders												
Epilepsy	**0.26**	**0.18, 0.34**	A	0.26	-0.03, 0.60	0.68	**0.30**	**0.24, 0.37**	**A**	**0.30**	**0.17, 0.53**	**0.67**

					C	1.00	-1.00, 1.00	0.21					C	0.76	-1.00, 1.00	0.00
					E	0.15	-0.22, 0.48	0.11					E	0.31	0.07, 0.54	0.33
					A	0.11	-1.00, 0.49	0.38								
	Migraine	**0.15**	**0.07, 0.23**		C	1.00	1.00, 1.00	0.51								
					E	0.07	-0.31, 0.42	0.11								
					A	0.15	-0.19, 0.47	0.63								
	Mixed headache	**0.14**	**0.06, 0.21**		C	1.00	1.00, 1.00	0.30								
					E	0.05	-0.29, 0.37	0.08								
Immunological disorders																
					A	0.03	-0.01, 0.21	0.16					A	0.09	-0.02, 0.20	0.71
	Asthma	**0.15**	**0.10, 0.18**		C	0.95	0.94, 0.96	0.86		**0.09**	**0.06, 0.13**		C	1.00	-1.00, 1.00	0.28
					E	-0.03	-0.27, 0.27	-0.02					E	0.00	-0.17, 0.17	0.01
Gastrointestinal disorders																
					A	0.19	-0.04, 0.45	0.73					**A**	**0.17**	**0.04, 0.29**	**0.80**
	Constipation	**0.16**	**0.10, 0.21**		C	1.00	-1.00, 1.00	0.31		**0.13**	**0.09, 0.17**		C	0.98	-1.00, 1.00	0.00
					E	-0.02	-0.27, 0.23	-0.03					E	0.07	-0.06, 0.22	0.20
					**A**	**0.21**	**0.02, 0.44**	**0.86**					**A**	**0.17**	**0.07, 0.28**	**0.93**
	Mixed FGIDs	**0.16**	**0.11, 0.21**		C	1.00	-1.00, 1.00	0.23		**0.12**	**0.08, 0.16**		C	0.78	-1.00, 1.00	0.00
					E	-0.07	-0.08, 0.16	-0.09					E	0.02	-0.00, 0.17	0.07

Physical health conditions in this study did not include the data from primary care health services.

Abbreviations: A, additive genetic variance; ADHD, attention-deficit/hyperactivity disorder; C, shared environmental variance; E, nonshared environmental variance; FGIDs, functional gastrointestinal disorders

Bold values: statistically significant

Table S6. Bivariate twin model of clinical autism and physical health conditions fit statistics

Model	−2LL	Parameters	*df*	AIC	Comparison model	Δ*χ2*	Δ*df*	*p*
Neurological disorders								
Epilepsy								
Fully saturated	5745.40	20	41368	-76990.60	NA	NA	NA	NA
ACE	5767.82	11	41381	-76994.18	Fully saturated	22.42	13	0.049
AE	5767.82	8	41384	-77000.18	ACE	0.00	3	1.000
CE	5826.02	8	41384	-76941.98	ACE	58.20	3	<0.001
E	6071.29	5	41387	-76702.71	ACE	303.48	6	<0.001
Immunological disorders								
Asthma								
Fully saturated	18023.33	20	41368	-64712.67	NA	NA	NA	NA
ACE	18053.61	11	41381	-64708.39	Fully saturated	30.29	13	0.004
AE	18063.48	8	41384	-64704.52	ACE	9.86	3	0.020
CE	18253.88	8	41384	-64514.12	ACE	200.27	3	<0.001
E	19646.57	5	41387	-63127.43	ACE	1592.96	6	<0.001
Coeliac disease								
Fully saturated	5231.88	20	41368	-77504.12	NA	NA	NA	NA
ACE	5230.12	11	41381	-77531.88	Fully saturated	-1.75	13	1.000
AE	5239.04	8	41384	-77528.97	ACE	8.91	3	0.031
CE	5318.31	8	41384	-77449.69	ACE	88.19	3	<0.001
E	5864.97	5	41387	-76909.03	ACE	634.85	6	<0.001
Gastrointestinal disorders								
Constipation								
Fully saturated	12499.09	20	41368	-70236.91	NA	NA	NA	NA
ACE	12528.91	11	41381	-70233.10	Fully saturated	29.82	13	0.005
AE	12529.02	8	41384	-70238.98	ACE	0.12	3	0.990
CE	12602.14	8	41384	-70165.86	ACE	73.24	3	<0.001
E	12935.16	5	41387	-69838.84	ACE	406.25	6	<0.001
Functional diarrhea								

	Fully saturated	4483.75	20	41368	-78252.25	NA	NA	NA	NA
	ACE	4518.13	11	41381	-78243.87	Fully saturated	34.38	13	0.001
	AE	4518.33	8	41384	-78249.67	ACE	0.20	3	0.977
	CE	4618.07	8	41384	-78149.93	ACE	99.94	3	<0.001
	E	4928.64	5	41387	-77845.36	ACE	410.51	6	<0.001
	Mixed FGIDs								
	Fully saturated	13224.49	20	41368	-69511.51	NA	NA	NA	NA
	ACE	13252.66	11	41381	-69509.34	Fully saturated	28.17	13	0.009
	AE	13252.69	8	41384	-69515.31	ACE	0.03	3	0.999
	CE	13337.88	8	41384	-69430.12	ACE	85.22	3	<0.001
	E	13725.48	5	41387	-69048.52	ACE	472.82	6	<0.001

Physical health conditions in this study did not include the data from primary care health services.

Abbreviations: −2LL, −2*log-likelihood; A, additive genetic component; AIC, Akaike Information Criteria; C, shared environmental component; E, nonshared environmental component; Eq, equate; Mixed FGIDs, mixed functional gastrointestinal disorders; NA, not applicable

Table S7. Bivariate twin model of subclinical autism and physical health conditions fit statistics

Model		−2LL	Parameters	*df*	AIC	Comparison model	Δ$\chi 2$	Δ*df*	*p*
Neurological disorders									
	Epilepsy								
	Fully saturated	8999.74	20	41368	-72366.26	NA	NA	NA	NA
	ACE	8875.23	11	41381	-72516.77	Fully saturated	-124.51	13	1.000
	AE	8875.23	8	41384	-72522.77	ACE	0.00	3	1.000
	CE	8961.11	8	41384	-72436.89	ACE	85.88	3	<0.001
	E	9333.76	5	41387	-72070.24	ACE	458.54	6	<0.001
Immunological disorders									
	Asthma								
	Fully saturated	21158.22	20	41368	-60207.77	NA	NA	NA	NA
	ACE	21184.59	11	41381	-60207.41	Fully saturated	26.37	13	0.015
	AE	21192.76	8	41384	-60205.24	ACE	8.16	3	0.043
	CE	21410.54	8	41384	-59987.46	ACE	225.94	3	<0.001
	E	22925.16	5	41387	-58478.84	ACE	1740.57	6	<0.001
Gastrointestinal disorders									
	Constipation								
	Fully saturated	15603.82	20	41368	-65762.19	NA	NA	NA	NA
	ACE	15636.63	11	41381	-65755.36	Fully saturated	32.82	13	0.002
	AE	15637.07	8	41384	-65760.93	ACE	0.44	3	0.933
	CE	15729.92	8	41384	-65668.08	ACE	93.29	3	< 0.001
	E	16200.04	5	41387	-65203.96	ACE	563.40	6	< 0.001
	Functional diarrhea								
	Fully saturated	7645.74	20	41368	-73720.25	NA	NA	NA	NA
	ACE	7654.73	11	41381	-73737.27	Fully saturated	8.99	13	0.774
	AE	7655.15	8	41384	-73742.85	ACE	0.41	3	0.938
	CE	7737.11	8	41384	-73660.90	ACE	82.37	3	<0.001
	E	8227.18	5	41387	-73176.82	ACE	572.44	6	<0.001
	Mixed FGIDs								

	Fully saturated	16339.72	20	41368	-65026.28	NA	NA	NA	NA
	ACE	16372.49	11	41381	-65019.51	Fully saturated	32.77	13	0.002
	AE	16372.73	8	41384	-65025.27	ACE	0.23	3	0.972
	CE	16476.94	8	41384	-64921.06	ACE	104.45	3	<0.001
	E	17001.94	5	41387	-64402.06	ACE	629.45	6	<0.001

Physical health conditions in this study did not include the data from primary care health services.

Abbreviations: −2LL, −2*log-likelihood; A, additive genetic component; AIC, Akaike Information Criteria; C, shared environmental component; E, nonshared environmental component; Eq, equate; Mixed FGIDs, mixed functional gastrointestinal disorders; NA, not applicable

Table S8. Bivariate twin model of clinical ADHD and physical health conditions fit statistics

Model	−2LL	Parameters	*df*	AIC	Comparison model	Δ*χ2*	Δ*df*	*p*
Neurological disorders								
Epilepsy								
Fully saturated	9280.38	20	41368	-73455.62	NA	NA	NA	NA
ACE	9299.88	11	41381	-73462.12	Fully saturated	19.49	13	0.109
AE	9302.25	8	41384	-73465.75	ACE	2.37	3	0.499
CE	9421.34	8	41384	-73346.66	ACE	121.46	3	<0.001
E	10094.64	5	41387	-72679.36	ACE	794.76	6	<0.001
Migraine								
Fully saturated	10100.13	20	41368	-72635.88	NA	NA	NA	NA
ACE	10122.30	11	41381	-72639.70	Fully saturated	22.17	13	0.053
AE	10125.35	8	41384	-72642.65	ACE	3.05	3	0.384
CE	10240.29	8	41384	-72527.72	ACE	117.99	3	<0.001
E	10885.59	5	41387	-71888.41	ACE	763.29	6	<0.001
Mixed headache								
Fully saturated	11072.10	20	41368	-71663.91	NA	NA	NA	NA
ACE	11116.01	11	41381	-71645.99	Fully saturated	43.92	13	<0.001
AE	11118.42	8	41384	-71649.58	ACE	2.41	3	0.492
CE	11239.33	8	41384	-71528.67	ACE	123.32	3	<0.001
E	11899.57	5	41387	-70874.43	ACE	783.56	6	<0.001
Immunological disorders								
Asthma								
Fully saturated	21507.59	20	41368	-61228.41	NA	NA	NA	NA
ACE	21530.83	11	41381	-61231.17	Fully saturated	23.24	13	0.039
AE	21545.26	8	41384	-61222.74	ACE	14.43	3	0.002
CE	21792.33	8	41384	-60975.67	ACE	261.50	3	<0.001
E	23615.84	5	41387	-59158.16	ACE	2085.00	6	<0.001
Gastrointestinal disorders								
Constipation								

	Fully saturated	15988.57	20	41368	-66747.43	NA	NA	NA	NA
	ACE	16010.12	11	41381	-66751.88	Fully saturated	21.55	13	0.063
	AE	16012.67	8	41384	-66755.33	ACE	2.55	3	0.466
	CE	16144.16	8	41384	-66623.84	ACE	134.04	3	<0.001
	E	16915.33	5	41387	-65858.67	ACE	905.21	6	<0.001
	Mixed FGIDs								
	Fully saturated	16719.20	20	41368	-66016.80	NA	NA	NA	NA
	ACE	16738.90	11	41381	-66023.11	Fully saturated	19.70	13	0.103
	AE	16741.28	8	41384	-66026.73	ACE	2.38	3	0.497
	CE	16884.76	8	41384	-65883.24	ACE	145.86	3	<0.001
	E	17712.44	5	41387	-65061.56	ACE	973.54	6	<0.001

Physical health conditions in this study did not include the data from primary care health services.

Abbreviations: −2LL, −2*log-likelihood; A, additive genetic component; AIC, Akaike Information Criteria; C, shared environmental component; E, nonshared environmental component; Eq, equate; Mixed FGIDs, mixed functional gastrointestinal disorders; NA, not applicable

Table S9. Bivariate twin model of subclinical ADHD and physical health conditions fit statistics

Model		−2LL	Parameters	*df*	AIC	Comparison model	Δ*χ2*	Δ*df*	*p*
Neurological disorders									
	Epilepsy								
	Fully saturated	16378.73	20	41368	-65859.27	NA	NA	NA	NA
	ACE	16426.30	11	41381	-65837.70	Fully saturated	47.57	13	<0.001
	AE	16426.30	8	41384	-65843.70	ACE	0.00	3	0.000
	CE	16604.98	8	41384	-65665.02	ACE	178.68	3	<0.001
	E	17136.47	5	41387	-65139.53	ACE	710.16	6	<0.001
Immunological disorders									
	Asthma								
	Fully saturated	28641.84	20	41368	-53596.16	NA	NA	NA	NA
	ACE	28710.10	11	41381	-53553.90	Fully saturated	68.26	13	<0.001
	AE	28718.90	8	41384	-53551.10	ACE	8.80	3	0.032
	CE	29026.36	8	41384	-53243.64	ACE	316.26	3	<0.001
	E	30706.36	5	41387	-51569.64	ACE	1996.25	6	<0.001
Gastrointestinal disorders									
	Constipation								
	Fully saturated	23112.60	20	41368	-59125.39	NA	NA	NA	NA
	ACE	23174.14	11	41381	-59089.86	Fully saturated	61.53	13	<0.001
	AE	23174.14	8	41384	-59095.86	ACE	0.00	3	1.000
	CE	23362.97	8	41384	-58907.03	ACE	188.83	3	<0.001
	E	23988.34	5	41387	-58287.66	ACE	814.20	6	<0.001
	Mixed FGIDs								
	Fully saturated	23846.95	20	41368	-58391.05	NA	NA	NA	NA
	ACE	23909.02	11	41381	-58354.98	Fully saturated	62.07	13	<0.001
	AE	23909.02	8	41384	-58360.98	ACE	0.00	3	1.000
	CE	24109.60	8	41384	-58160.40	ACE	200.58	3	<0.001
	E	24791.72	5	41387	-57484.29	ACE	882.69	6	<0.001

Physical health conditions in this study did not include the data from primary care health services.
Abbreviations: −2LL, −2*log-likelihood; A, additive genetic component; AIC, Akaike Information Criteria; C, shared environmental component; E, nonshared environmental component; Eq, equate; Mixed FGIDs, mixed functional gastrointestinal disorders; NA, not applicable

Table S10. Etiological component contributing to phenotypic correlation between subclinical autism/ADHD (clinical autism/ADHD excluded) and physical health conditions

Co-occurring physical health conditions	Subclinical autism						Subclinical ADHD					
	Phenotypic Correlation			Etiological components			Phenotypic Correlation			Etiological components		
				Correlations		Propor -tions				Correlations		Propor -tions
	r	95% CI		*r*	95% CI		*r*	95% CI		*r*	95% CI	
Neurological disorders												
Epilepsy	**0.33**	**0.25, 0.41**	A	0.26	-0.03, 0.51	0.54	**0.24**	**0.17, 0.31**	**A**	**0.20**	**0.04, 0.45**	**0.53**
			C	0.96	-1.00, 1.00	0			C	0.60	-0.64, 1.00	0
			**E**	**0.54**	**0.27, 0.95**	**0.46**			E	0.31	0.07, 0.55	0.47
Immunological disorders												
Asthma	**0.11**	**0.06, 0.16**	A	0.14	-0.07, 0.35	0.97	**0.05**	**0.01, 0.08**	A	0.01	-0.01, 0.13	0.09
			C	-1.00	-1.00, -1.00	-0.31			C	1.00	-0.95, 1.00	0.42
			E	0.20	-0.09, 0.48	0.34			E	0.09	-0.09, 0.27	0.49
Gastrointestinal disorders												
Constipation	**0.18**	**0.12, 0.23**	A	0.17	-0.12, 0.39	0.56	**0.09**	**0.05, 0.14**	A	0.12	-0.03, 0.26	0.73
			C	1.00	-1.00, 1.00	0.29			C	0.99	-0.39, 1.00	0
			E	0.08	-0.19, 0.29	0.15			E	0.06	-0.09, 0.21	0.27
Functional diarrhea	**0.12**	**0.00, 0.24**	A	0.17	-0.16, 0.31	1.12						
			C	-1.00	-1.00, 1.00	-0.16						
			E	0.04	-0.45, 0.69	0.03						
Mixed FGIDs	**0.16**	**0.14, 0.22**	A	0.15	-0.11, 0.34	0.55	**0.08**	**0.05, 0.13**	**A**	**0.11**	**0.07, 0.20**	**0.81**
			C	1.00	-1.00, 1.00	0.28			C	0.90	-0.92, 1.00	0
			E	0.09	-0.12, 0.30	0.17			E	0.04	-0.10, 0.09	0.19

Physical health conditions in this study did not include the data from primary care health services.

Abbreviations: A, additive genetic variance; ADHD, attention-deficit/hyperactivity disorder; C, shared environmental variance; E, nonshared environmental variance; FGIDs, functional gastrointestinal disorders

Bold values: statistically significant
