# Supplementary material for: Genetic and environmental contributions to co-occurring physical health conditions in autism spectrum condition and attention-deficit/hyperactivity disorder
Source: Mol Autism. 2023 Apr 21;14:17. doi: 10.1186/s13229-023-00548-3 (PMC10122407; doi:10.1186/s13229-023-00548-3)
Supplement: Supplementary file 2 — Additional file 2. The English version of the Autism–Tics, ADHD, and Other Comorbidities inventory (A-TAC). [file 13229_2023_548_MOESM2_ESM.pdf]

A-TAC: FV
Child and adolescent version

This questionnaire is in particular detail focused on a number of abilities and behaviours in children. All children are different from one another. This means that their abilities in various areas as well as their conduct and behaviour vary a great deal.

To gain as complete a picture as possible of your child, we ask you to answer a considerable number of questions.

Naturally, children function in different ways at different ages. State your perception of your child's functioning as compared to his or her peers. If your child has had a certain problem or specific characteristic during *any period of life*, answer the question with "yes" even if the problem or characteristic is no longer present.

Name of child/youth: ____

Date of birth/personal identity number: ______________________________

Age: __________ **Sex:** ___________

Date of interview: __________________________

Informant (the person answering the questions): _______________________

Informant's relationship to the
child/youth (i.e. mother, father, etc): __________________________________

Henrik Anckarsäter Björn Kadesjö
Carina Gillberg Maria Råstam
Christopher Gillberg Ola Ståhlberg

Developed at the Department of Child and Adolescent Psychiatry, Göteborg University

English translation: Sheila Allein, Henrik Anckarsäter, Christopher Gillberg, Ola Ståhlberg

**A. Motor control**	The essential aspect of each question is whether the problem/peculiarity has been **pronounced compared to peers during any period of life**	Yes	Yes, to some extent	No
1	Does he/she have problems coordinating movements smoothly?	☐	☐	☐
	**If "Yes" or "Yes, to some extent" to this question:**			
A1	Is he/she clumsy?	☐	☐	☐
A2	Is he/she fumbling?	☐	☐	☐
A3	Does he/she have balance problems?	☐	☐	☐
A4	Does he/she easily stumble and fall?	☐	☐	☐
A5	Have the peculiarities or problems relating to motor control caused significant impairment in school, among peers or at home?	☐	☐	☐
A6	Do the peculiarities or problems relating to motor control cause him/her significant suffering?	☐	☐	☐
A7	At what age did the peculiarities or problems relating to motor control commence?	Age:		
A8	Are they still present?	Yes ☐ No ☐		

**B. Perception**	The essential aspect of each question is whether the problem/peculiarity has been **pronounced compared to peers during any period of life**	Yes	Yes, to some extent	No
2	Does he/she seem disturbed by height differences such as in connection with climbing stairs etc.?	☐	☐	☐
3	Does he/she have difficulty judging distance or size?	☐	☐	☐
4	Is he/she oversensitive to touch or tight clothing?	☐	☐	☐
5	Is he/she particularly sensitive to certain sounds/noise?	☐	☐	☐
6	Is he/she particularly sensitive to certain flavours, smells, or consistencies?	☐	☐	☐
	**If "Yes" or "Yes, to some extent" to any of these questions:**			
B1	Does he/she have difficulty comprehending orientation and spatial directions, e.g. puts clothes on backwards?	☐	☐	☐
B2	Does he/she often bump into other people?	☐	☐	☐
B3	Does he/she have poor concepts of time?	☐	☐	☐
B4	Does he/she have difficulty imitating other people's movements?	☐	☐	☐
B5	Does he/she have difficulty recognizing people?	☐	☐	☐
B6	Have the peculiarities or problems relating to perception caused significant impairment in school, among peers or at home?	☐	☐	☐
B7	Do the peculiarities or problems relating to perception cause him/her significant suffering?	☐	☐	☐
B8	At what age did the peculiarities or problems relating to perception commence?	Age:		
B9	Are they still present?	Yes ☐ No ☐		

C. Concentration and attention	The essential aspect of each question is whether the problem/peculiarity has been **pronounced compared to peers during any period of life**	Yes	Yes, to some extent	No
7	Does he/she often fail to pay close attention to details or make careless mistakes in schoolwork, or other activities?	☐	☐	☐
8	Does he/she often have difficulty sustaining attention in tasks or play activities?	☐	☐	☐
9	Does he/she often seem not to listen when spoken to directly?	☐	☐	☐
10	Does he/she have difficulty following instructions and finishing tasks?	☐	☐	☐
11	Does he/she often have difficulty organizing tasks and activities?	☐	☐	☐
12	Does he/she often avoid tasks that require sustained mental effort (such as homework)?	☐	☐	☐
13	Does he/she often lose things?	☐	☐	☐
14	Is he/she easily distracted or disturbed?	☐	☐	☐
15	Is he/she often forgetful in daily activities?	☐	☐	☐
	**If "Yes" or "Yes, to some extent" to any of these questions:**			
C1	Does he/she have difficulty getting started on tasks/activities?	☐	☐	☐
C2	Does he/she have difficulty completing tasks/activities?	☐	☐	☐
C3	Have the peculiarities or problems relating to concentration and attention caused significant impairment in school, among peers or at home?	☐	☐	☐
C4	Do the peculiarities or problems relating to concentration and attention cause him/her significant suffering?	☐	☐	☐
C5	At what age did the peculiarities or problems relating to concentration and attention commence?	Age:		
C6	Are they still present?	Yes ☐ No ☐		

D. Impulsiveness and activity	The essential aspect of each question is whether the problem/peculiarity has been **pronounced compared to peers during any period of life**	Yes	Yes, to some extent	No
16	Does he/she have difficulties keeping his/her hands and feet still or can he/she not stay seated?	☐	☐	☐
17	Does he/she get up and move about in class or in other situations when he/she is supposed to remain seated?	☐	☐	☐
18	Does he/she often run around or climb excessively compared to peers?	☐	☐	☐
19	Does he/she have difficulty playing calmly and quietly?	☐	☐	☐
20	Does he/she often act as though he/she had "ants in his/her pants", i.e. being unable to stay still?	☐	☐	☐
21	Does he/she talk constantly?	☐	☐	☐
22	Does he/she often blurt out answers to questions before they are completed?	☐	☐	☐
23	Does he/she have difficulty waiting for his/her turn?	☐	☐	☐
24	Does he/she often interrupt, or intrude on, others?	☐	☐	☐
25	Does he/she easily get bored?	☐	☐	☐
	**If "Yes" or "Yes, to some extent" to any of these questions:**			
D1	Is he/she unusually intrepid in physically dangerous situations?	☐	☐	☐
D2	Have the peculiarities or problems relating to impulsiveness and activity caused significant impairment in school, among peers or at home?	☐	☐	☐
D3	Do the peculiarities or problems impulsiveness and activity cause him/her significant suffering?	☐	☐	☐
D4	At what age did the peculiarities or problems relating to impulsiveness and activity commence?	Age:		
D5	Are they still present?	Yes ☐ No ☐		

**E. Learning**		The essential aspect of each question is whether the problem/peculiarity has been **pronounced compared to peers during any period of life**	Yes	Yes, to some extent	No
26	Has he/she had more difficulties than expected acquiring reading skills?		☐	☐	☐
27	Is learning slow and laborious for him/her?		☐	☐	☐
28	Does he/she have difficulties with basic maths?		☐	☐	☐
	**If "Yes" or "Yes, to some extent" to any of these questions:**				
E1	Is he/she a slow reader?		☐	☐	☐
E2	Does he/she dislike reading (e.g. does he/she avoid reading books)?		☐	☐	☐
E3	Does he/she have difficulties solving maths problems, which require him/her to read written material?		☐	☐	☐
E4	Does he/she have difficulties understanding or using abstract terms?		☐	☐	☐
E5	Does he/she have difficulties spelling?		☐	☐	☐
E6	Does he/she get special education in school?		☐	☐	☐
E7	Have peculiarities or problems relating to learning caused significant impairment in school, among peers or at home?		☐	☐	☐
E8	Do the peculiarities or problems relating to learning cause him/her significant suffering?		☐	☐	☐
E9	At what age did the peculiarities or problems relating to learning commence?		Age:		
E10	Are they still present?		Yes ☐ No ☐		

**F. Planning and organizing tasks**		The essential aspect of each question is whether the problem/peculiarity has been **pronounced compared to peers during any period of life**	Yes	Yes, to some extent	No
29	Does he/she have difficulty shifting plan or strategy when this is required?		☐	☐	☐
30	Does he/she have difficulty keeping things in order around him/her?		☐	☐	☐
	**If "Yes" or "Yes, to some extent" to any of these questions:**				
F1	Does he/she have difficulties understanding consequences of his/her own actions?		☐	☐	☐
F2	Is he/she dependent and very much in need of support?		☐	☐	☐
F3	Does he/she find it difficult to take care of his/her personal hygiene, his/her clothes, and the like?		☐	☐	☐
F4	Does he/she have difficulties in postponing rewards until later and finding the meaning in things that are not immediately rewarding?		☐	☐	☐
F5	Does he/she experience simple, everyday activities as tiring or energy consuming?		☐	☐	☐
F6	Have peculiarities or problems relating to planning and organizing tasks caused significant impairment in school, among peers or at home?		☐	☐	☐
F7	Do the peculiarities or problems relating to planning and organizing tasks cause him/her significant suffering?		☐	☐	☐
F8	At what age did the peculiarities or problems relating to planning and organizing tasks commence?		Age:		
F9	Are they still present?		Yes ☐ No ☐		

G. Memory		The essential aspect of each question is whether the problem/peculiarity has been **pronounced compared to peers during any period of life**	Yes	Yes, to some extent	No
31	Does he/she have difficulties remembering where he/she put things?		☐	☐	☐
32	Does he/she have difficulties remembering long or multiple-step instructions?		☐	☐	☐
33	Does he/she have difficulties learning rhymes, songs, multiplication tables etc by heart?		☐	☐	☐
	**If "Yes" or "Yes, to some extent" to any of these questions:**				
G1	Does he/she have difficulties remembering information about personal data, such as date of birth, home address etc.?		☐	☐	☐
G2	Does he/she have difficulties remembering the names of people around him?		☐	☐	☐
G3	Does he/she have difficulties remembering the names of weekdays, months and seasons?		☐	☐	☐
G4	Does he/she have difficulties remembering non-personal facts learned at school (e.g. historic events, chemical formulas etc.)?		☐	☐	☐
G5	Does he/she have difficulty remembering specific events that he/she recently experienced, e.g. what happened during the day or, what he/she ate a few hours ago etc.?		☐	☐	☐
G6	Does he/she have difficulties remembering events that occurred some time ago, such as what happened on a trip, what Christmas presents he/she got etc.?		☐	☐	☐
G7	Does he/she have difficulties remembering appointments with peers or what homework he/she has?		☐	☐	☐
G8	Does he/she have difficulties learning the rules of new games, sports etc.?		☐	☐	☐
G9	Have peculiarities or problems relating to memory caused significant impairment in school, among peers or at home?		☐	☐	☐
G10	Do the peculiarities or problems relating to memory cause him/her significant suffering?		☐	☐	☐
G11	At what age did the peculiarities or problems relating to memory commence?		Age:		
G12	Are they still present?		Yes ☐ No ☐		

H. Language		The essential aspect of each question is whether the problems/characteristics has been **pronounced compared to peers during any period of life**	Yes	Yes, to some extent	No
34	Was his/her language development delayed or doesn't he/she speak at all?		☐	☐	☐
35	Does he/she have difficulties sustaining a conversation?		☐	☐	☐
36	Does he/she like to repeat words and expressions or does he/she use words in a way that other people find strange?		☐	☐	☐
37	Does he/she have difficulties with games of make-believe or does he/she imitate others considerably less than other children?		☐	☐	☐
38	Does he/she talk in too high a pitch or too quietly?		☐	☐	☐
39	Does he/she have difficulties keeping "on track" when telling other people something?		☐	☐	☐
	**If "Yes" or "Yes, to some extent" to any of these questions:**				
H1	Does he/she have difficulties expressing him/herself in whole sentences?		☐	☐	☐
H2	Does he/she speak in a monotonous or strange voice?		☐	☐	☐
H3	Does he/she have difficulties explaining/recounting his/her experiences to other people?		☐	☐	☐
H4	Does he/she have difficulties explaining what he/she wants?		☐	☐	☐
H5	Does he/she have difficulties speaking fluently, without any pauses?		☐	☐	☐
H6	Does he/she have difficulties pronouncing complex words?		☐	☐	☐

H7	Does he/she explain emotions verbally so that other people have difficulty understanding what he/she means?	☐	☐	☐
H8	Does he/she use strange neologisms, old-fashioned words, or too elegant words?	☐	☐	☐
H9	Does he/she speak so rapidly that it is difficult to comprehend what he/she is saying?	☐	☐	☐
H10	Have peculiarities or problems relating to language caused significant impairment in school, among peers or at home?	☐	☐	☐
H11	Do the peculiarities or problems relating to language cause him/her significant suffering?	☐	☐	☐
H12	At what age did the peculiarities or problems relating to language commence?	Age:		
H13	Are they still present?	Yes ☐ No ☐		

I. Social interaction	The essential aspect of each question is whether the problem/peculiarity has been **pronounced compared to peers during any period of life**	Yes	Yes, to some extent	No
40	Does he/she have difficulties expressing emotions and reactions with facial gestures, prosody, or body language?	☐	☐	☐
41	Does he/she exhibit considerable difficulties interacting with peers?	☐	☐	☐
42	Is he/she uninterested in sharing joy, interests, and activities with others?	☐	☐	☐
43	Can he/she only be with other people on his/her terms?	☐	☐	☐
44	Does he/she have difficulties behaving as expected by peers?	☐	☐	☐
45	Do other people easily influence him/her?	☐	☐	☐
	**If ”Yes” or ”Yes, to some extent” to any of these questions:**			
I1	Is he/she self-centred?	☐	☐	☐
I2	Is he/she perceived as different, odd, or eccentric by peers?	☐	☐	☐
I3	Does he/she have difficulty understanding other people's social cues, e.g., facial expressions, gestures, tone of voice, or body language?	☐	☐	☐
I4	Does he/she have difficulty understanding the feelings of other people?	☐	☐	☐
I5	Does he/she have difficulty showing other people respect?	☐	☐	☐
I6	Does he/she get overly excited when there are a lot of people around?	☐	☐	☐
I7	Does he/she often leave in the middle of a conversation, or abruptly change the topic of a conversation?	☐	☐	☐
I8	Does he/she have difficulty realising how to behave in different social situations?	☐	☐	☐
I9	Does he/she inadvertently make a fool of him/herself or does he/she make embarrassing remarks?	☐	☐	☐
I10	Does he/she often seem to lack common sense?	☐	☐	☐
I11	Does he/she have difficulty with eye contact?	☐	☐	☐
I12	Does he/she think that relationships are not very important or does he/she prefer to be on his/her own?	☐	☐	☐
I13	Is his/her body language awkward, gauche, clumsy, strange or unusual?	☐	☐	☐
I14	Does he/she have difficulty interpreting what is conveyed through eye contact?	☐	☐	☐
I15	Is his/her gaze stiff, strange, peculiar, abnormal or odd?	☐	☐	☐
I16	Have peculiarities or problems relating to social interaction caused significant impairment in school, among peers or at home?	☐	☐	☐
I17	Do the peculiarities or problems relating to social interaction cause him/her significant suffering?	☐	☐	☐
I18	At what age did the peculiarities or problems relating to social interaction commence?	Age:		
I19	Are they still present?	Yes ☐ No ☐		

**J. Flexibility**	The essential aspect of each question is whether the problem/peculiarity has been **pronounced compared to peers during any period of life**	Yes	Yes, to some extent	No
46	Does he/she get absorbed by his/her interests in such a way as being repetitive or too intense?	☐	☐	☐
47	Does he/she get absorbed by routines in such a way as to produce problems for him/herself or others?	☐	☐	☐
48	Has he/she ever engaged in strange hand movements or toe-walking when he/she was happy or upset?	☐	☐	☐
49	Does he/she get obsessed with details?	☐	☐	☐
50	Does he/she dislike changes in daily routines?	☐	☐	☐
	**If ″Yes″ or ″Yes, to some extent″ to any of these questions:**			
J1	Have peculiarities or problems relating to flexibility caused significant impairment in school, among peers or at home?	☐	☐	☐
J2	Do the peculiarities or problems relating to flexibility cause him/her significant suffering?	☐	☐	☐
J3	At what age did the peculiarities or problems relating to flexibility commence?	Age:		
J4	Are they still present?	Yes ☐ No ☐		

**K. Tics**	The essential aspect of each question is whether the problem/peculiarity has been **pronounced compared to peers during any period of life**	Yes	Yes, to some extent	No
51	Has he/she during any period of life made involuntary sounds such as throat clearing, sneezing, swallowing, barking, or shouting?	☐	☐	☐
52	Has he/she during any period of life made involuntary facial grimaces or body movements?	☐	☐	☐
53	Does he/she make a lot of noise, e.g. whistle, hum, mumble?	☐	☐	☐
	**If ″Yes″ or ″Yes, to some extent″ to any of these questions:**			
K1	Does he/she curse or use dirty words in an exaggerated way?	☐	☐	☐
K2	Have peculiarities or problems relating to tics caused significant impairment in school, among peers or at home?	☐	☐	☐
K3	Do the peculiarities or problems relating to tics cause him/her significant suffering?	☐	☐	☐
K4	At what age did the peculiarities or problems relating to tics commence?	Age:		
K5	Are they still present?	Yes ☐ No ☐		

**L. Compulsions**	The essential aspect of each question is whether the problem/peculiarity has been **pronounced compared to peers during any period of life**	Yes	Yes, to some extent	No
54	Does he/she have obsessive thoughts, i.e. thoughts that recur over and over again and that he/she cannot stop, for example about dirt, contagion or that something terrible will happen?	☐	☐	☐
55	Does he/she have compulsive behaviours such as washing his/her hands, touching things, checking on things, repeating things or procedures, arranging or ordering things, or counting?	☐	☐	☐
	**If ″Yes″ or ″Yes, to some extent″ to any of these questions:**			
L1	Have peculiarities or problems relating to compulsions caused significant impairment in school, among peers or at home?	☐	☐	☐

L2	Do the peculiarities or problems relating to compulsions cause him/her significant suffering?			
L3	At what age did the peculiarities or problems relating to compulsions commence?	Age:		
L4	Are they still present?	Yes No		

M. Eating habits	The essential aspect of each question is whether the problem/peculiarity has been **pronounced compared to peers during any period of life**	Yes	Yes, to some extent	No
56	Has he/she ever failed to gain enough weight for more than a year or been underweight?			
57	Has he/she appeared to be fearful of gaining weight or becoming fat?			
	**If "Yes" or "Yes, to some extent" to any of these questions:**			
M1	Has he/she dieted hard enough to cause underweight or no weight gain for any length of time?			
M2	Has he/she exercised excessively or has he/she been overly interested in his/her physical appearance?			
M3	Females only: Has she failed to menstruate for at least 3 months due to weight loss?			
M4	Has he/she had periods of overeating followed by vomiting?			
M5	Has he/she ever tried to lose weight in spite of already being thin?			
M6	Has he/she ever had anorexia nervosa?			
M7	Have peculiarities or problems relating to eating caused significant impairment in school, among peers or at home?			
M8	Do the peculiarities or problems relating to eating cause him/her significant suffering?			
M9	At what age did the peculiarities or problems relating to eating commence?	Age:		
M10	Are they still present?	Yes No		

N. Separations	The essential aspect of each question is whether the problem/peculiarity has been **pronounced compared to peers during any period of life**	Yes	Yes, to some extent	No
58	Does he/she have difficulties functioning outside the family home?			
59	Does he/she often voice fears that family members may die or get hurt?			
60	Does he/she have an unreasonable fear of being alone or home alone?			
61	Does he/she have difficulty sleeping if family members are not around?			
62	Does he/she complain about recurring headaches, bellyaches, nausea or vomiting after being separated from loved ones?			
	**If "Yes" or "Yes, to some extent" to any of these questions:**			
N1	Does he/she have difficulty leaving home to go to school for fear of being separated from his/her family?			
N2	Does he/she have recurring nightmares about being separated from the family?			
N3	Does he/she react unusually strongly when friendships or other close relationships come, or are about to come, to an end?			
N4	Have peculiarities or problems relating to separation caused significant impairment in school, among peers or at home?			
N5	Do the peculiarities or problems relating to separation cause him/her significant suffering?			
N6	At what age did the peculiarities or problems relating to separation commence?	Age:		
N7	Are they still present?	Yes No		

**O+P. Defiance/Conduct**	The essential aspect of each question is whether the problem/peculiarity has been **pronounced compared to peers during any period of life**	Yes	Yes, to some extent	No
63	Has there ever been a time when he/she was so angry that he/she could not be reached?	☐	☐	☐
64	Does he/she often argue with adults?	☐	☐	☐
65	Does he/she often tease others by deliberately doing things that are perceived as provocative?	☐	☐	☐
66	Is he/she easily offended, or disturbed by others?	☐	☐	☐
67	Is he/she easily teased?	☐	☐	☐
68	Does he/she often lie or cheat?	☐	☐	☐
69	Has he/she ever engaged in shoplifting?	☐	☐	☐
70	Has he/she ever deliberately been physically cruel to anybody?	☐	☐	☐
71	Does he/she often get into fights?	☐	☐	☐
72	Does he/she steal things at home or away from home?	☐	☐	☐
	**If "Yes" or "Yes, to some extent" to any of these questions:**			
O1	Does he/she often lose his/her temper?	☐	☐	☐
O2	Does he/she often refuse to follow other directives from adults?	☐	☐	☐
O3	Is he/she often vindictive or cruel?	☐	☐	☐
O4	Does he/she often treat people close to him/her badly or without respect?	☐	☐	☐
O5	Does he/she often blame others for his/her own mistakes?	☐	☐	☐
P1	Does he/she often threaten, harass or humiliate others?	☐	☐	☐
P2	Is he/she cruel to bugs/insects?	☐	☐	☐
P3	Is he/she cruel to other animals?	☐	☐	☐
P4	Has he/she ever started a fire?	☐	☐	☐
P5	Has he/she ever sexually abused other children?	☐	☐	☐
P6	Has he/she ever been detained by the police?	☐	☐	☐
P7	Has he/she ever used a weapon that could cause serious physical harm?	☐	☐	☐
P8	Has he/she ever robbed anyone or else unlawfully acquired other people's property by means of direct threats?	☐	☐	☐
P9	Has he/she ever purposely attempted to destroy other people's property?	☐	☐	☐
P10	Has he/she ever broken into someone else's home, premises or car?	☐	☐	☐
P11	Is he/she often out late at night without consent (beginning before 13 years of age)?	☐	☐	☐
P12	Has he/she ever run away from home and spent the night elsewhere at least twice (or once if it was for an extended period of time)?	☐	☐	☐
P13	Is he/she often skipping school (beginning before 13 years of age)?	☐	☐	☐
OP14	Have peculiarities or problems relating to defiance/conduct caused significant impairment in school, among peers or at home?	☐	☐	☐
OP15	Do the peculiarities or problems relating to defiance/conduct cause him/her significant suffering?	☐	☐	☐
OP16	At what age did the peculiarities or problems relating to defiance/conduct commence?	Age:		
OP17	Are they still present?	Yes ☐ No ☐		

**Q. Anxiety**	The essential aspect of each question is whether the problem/peculiarity has been **pronounced compared to peers during any period of life**	Yes	Yes, to some extent	No
73	Does he/she have panic attacks with sudden strong fear or anxiety?	☐	☐	☐
74	Does he/she fear leaving the house alone, being in crowds, waiting in line or going on a bus or train?	☐	☐	☐
75	Is he/she often particularly nervous or anxious?	☐	☐	☐
	**If "Yes" or "Yes, to some extent" to any of these questions:**			
Q1	Is he/she extremely shy and reticent?	☐	☐	☐
Q2	Is he/she silent in situations where you are not expected to be silent?	☐	☐	☐
Q3	Is there anything he/she fears doing in front of other people, i.e. talking, eating or writing (excluding presentations of reports)?	☐	☐	☐
Q4	Have peculiarities or problems relating to anxiety caused significant impairment in school, among peers or at home?	☐	☐	☐
Q5	Do the peculiarities or problems relating to anxiety cause him/her significant suffering?	☐	☐	☐
Q6	At what age did the peculiarities or problems relating to anxiety commence?	Age:		
Q7	Are they still present?	Yes ☐ No ☐		

**R. Mood**	The essential aspect of each question is whether the problem/peculiarity has been **pronounced compared to peers during any period of life**	Yes	Yes, to some extent	No
76	Does he/she have poor self-confidence?	☐	☐	☐
77	Does he/she often complain about bellyaches, headaches, breathing difficulties or other bodily symptoms?	☐	☐	☐
78	Has he/she had recurrent episodes with extremely high activity level, talkativeness and flight of ideas?	☐	☐	☐
79	Does he/she have recurrent periods of obvious irritability?	☐	☐	☐
80	Does his/her self-confidence vary considerably across different situations?	☐	☐	☐
	**If "Yes" or "Yes, to some extent" to any of these questions:**			
R1	Does he/she often appear to be unhappy, sad, or depressed?	☐	☐	☐
R2	Does he/she often complain about a feeling of loneliness?	☐	☐	☐
R3	Does he/she often express a feeling of being worthless or inferior to peers?	☐	☐	☐
R4	Has there ever been a period when nothing could make him/her feel happy?	☐	☐	☐
R5	Has he/she been thinking of or talked about committing suicide?	☐	☐	☐
R6	Has he/she tried to commit suicide?	☐	☐	☐
R7	Has he/she often had a feeling of emptiness?	☐	☐	☐
R8	Does he/she feel that his/her qualities and talents are ignored by others?	☐	☐	☐
R9	Have peculiarities or problems relating to his/her mood caused significant impairment in school, among peers or at home?	☐	☐	☐
R10	Do the peculiarities or problems relating to his/her mood cause him/her significant suffering?	☐	☐	☐
R11	At what age did the peculiarities or problems relating to his/her mood commence?	Age:		
R12	Are they still present?	Yes ☐ No ☐		

**S. Concept of reality**	The essential aspect of each question is whether the problem/peculiarity has been **pronounced compared to peers during any period of life**	Yes	Yes, to some extent	No
81	Has he/she ever had visions or seen things that no one else could see?	☐	☐	☐
	**If ″Yes″ or ″Yes, to some extent″ to this question:**			
S1	Has he/she ever perceived him/herself as being followed or haunted by others even though this has not actually been the case?	☐	☐	☐
S2	Has he/she ever heard voices or sounds, which no one else could hear?	☐	☐	☐
S3	Have peculiarities or problems relating to concept of reality caused significant impairment in school, among peers or at home?	☐	☐	☐
S4	Do the peculiarities or problems relating to concept of reality cause him/her significant suffering?	☐	☐	☐
S5	At what age did the peculiarities or problems relating to concept of reality commence?	Age:		
S6	Are they still present?	Yes ☐ No ☐		

**T. Miscellaneous**	The essential aspect of each question is whether the problem/peculiarity has been **pronounced compared to peers during any period of life**	Yes	Yes, to some extent	No
82	Does he/she stutter?	☐	☐	☐
83	Is he/she or has she/she ever been bullied by other children in school?	☐	☐	☐
84	Has he/she ever been severely overweight?	☐	☐	☐
85	Does he/she often have sleeping problems?	☐	☐	☐
86	Does he/she often have nightmares?	☐	☐	☐
87	Does he/she often walk in his/her sleep or have nocturnal attacks when he/she cannot be ″reached″ or comforted?	☐	☐	☐
88	Has he/she ever deliberately hurt him/herself?	☐	☐	☐
89	Has he/she repeatedly and purposely hurt him/herself?	☐	☐	☐
90	Is there anything he/she fears excessively, such as flying, see blood, have an injection, heights, cramped rooms, or certain kind of animals or insects?	☐	☐	☐
91	Has he/she wet him/herself during daytime on several occasions after the age of 5?	☐	☐	☐
92	Has he/she soiled him/herself on several occasions after the age of 4 except in connection with gastro-intestinal infection?	☐	☐	☐
93	Does he/she smoke?	☐	☐	☐
94	Does he/she use any other form of tobacco?	☐	☐	☐
95	Has he/she ever used alcohol?	☐	☐	☐
96	Has he/she ever had a period after age 5 when he/she only wanted to eat particular types of food?	☐	☐	☐
